# Temperature and Rainfall Patterns Constrain the Multidimensional Rewilding of Global Forests

**DOI:** 10.1002/advs.202201144

**Published:** 2022-04-25

**Authors:** Guiyao Zhou, Xuhui Zhou, David J. Eldridge, Ximei Han, Yanjun Song, Ruiqiang Liu, Lingyan Zhou, Yanghui He, Zhenggang Du, Manuel Delgado‐Baquerizo

**Affiliations:** ^1^ Zhejiang Tiantong Forest Ecosystem National Observation and Research Station Center for Global Change and Ecological Forecasting School of Ecological and Environmental Sciences East China Normal University Shanghai 200241 China; ^2^ Northeast Asia Ecosystem Carbon Sink Research Center (NACC) Center for Ecological Research Key Laboratory of Sustainable Forest Ecosystem Management‐Ministry of Education School of Forestry Northeast Forestry University Harbin 150040 China; ^3^ Centre for Ecosystem Science School of Biological Earth and Environmental Sciences University of New South Wales Sydney New South Wales 2052 Australia; ^4^ Forest Ecology and Forest Management Group Wageningen University and Research P.O. Box 47 Wageningen 6700 AA the Netherlands; ^5^ Instituto de Recursos Naturales y Agrobiología de Sevilla (IRNAS) CSIC Av. Reina Mercedes 10 Sevilla E‐41012 Spain

**Keywords:** biodiversity‐ecosystem function, carbon sequestration, climate change, forest restoration, tradeoffs

## Abstract

The long‐term contribution of global forest restoration to support multiple dimensions of biodiversity and ecosystem function remains largely illusive across contrasting climates and forest types. This hampers the capacity to predict the future of forest rewilding under changing global climates. Here, 120 studies are synthesized across five continents, and it is found that forest restoration promotes multiple dimensions of biodiversity and ecosystem function such as soil fertility, plant biomass, microbial habitat, and carbon sequestration across contrasting climates and forest types. Based on global relationship between stand age and soil organic carbon stock, planting 350 million hectares of forest under the UN Bonn Challenge can sequester >30 Gt soil C in the surface 20 cm over the next century. However, these findings also indicate that predicted increases in temperature and reductions in precipitation can constrain the positive effects of forest rewilding on biodiversity and ecosystem function. Further, important tradeoffs are found in very old forests, with considerable disconnection between biodiversity and ecosystem function. Together, these findings provide evidence of the importance of the multidimensional rewilding of forests, suggesting that on‐going climatic changes may dampen the expectations of the positive effects of forest restoration on biodiversity and ecosystem function.

## Graphical abstract

Forest restoration globally promotes multiple dimensions of biodiversity and ecosystem function such as soil fertility, plant biomass, microbial habitat, and carbon sequestration across contrasting climates and forest types. However, increases in temperature and reductions in precipitation can constrain the positive effects of forest rewilding on biodiversity and multiple ecosystem function.

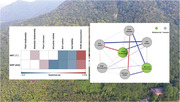

## 1 Introduction

Global concern over deforestation and climate change has spawned initiatives such as The Bonn Challenge,^[^ ^1^ ^]^ the New York Declaration on Forests, and related AFR100^[^ ^2^ ^]^ to reduce the global rate of deforestation^[^ ^3^ ^]^ and promote forest restoration. Forest restoration is seen as an effective strategy for protecting biodiversity, enhancing human well‐being, and providing effective solutions to the effects of changing climate.^[^ ^3^, ^4^, ^5^, ^6^ ^]^ Targets for forest restoration are to restore 350 million hectares of forests by 2030 to tackle climate change and enhance human well‐being.^[1]^ Increasing forest coverage by 1 billion hectares is expected to limit global warming to 1.5 °C by 2050.^[^ ^7^ ^]^ Yet, despite the enthusiasm surrounding forest restoration, the extent to which it will lead to long‐term global improvements in biodiversity and ecosystem function in forested systems is still largely unknown. This knowledge is important if we are to motivate governments and institutions to invest in global forest restoration and use natural‐based solutions to achieve global climate targets.^[^ ^3^, ^5^ ^]^

Our current understanding of the capacity of global forest rewilding to sustain long‐term biodiversity and ecosystem function is hampered by a poor understanding of the multiple dimensions of biodiversity and ecosystem function and the narrow local focus of most studies to date. For example, most restoration studies have focused on a limited suite of ecosystem attributes, such as plant biodiversity,^[^ ^8^ ^]^ plant biomass,^[^ ^9^ ^]^ carbon sequestration,^[^ ^5^ ^]^ and soil fertility,^[^ ^10^ ^]^ with limited focus on the multidimensional interconnections among these many attributes.^[^ ^11^, ^12^, ^13^ ^]^ This is problematic, because it overlooks the multiple dimensions of plant and soil biodiversity, and their interdependencies (i.e., multifunctionality), which can lead to both positive and negative long‐term outcomes for ecosystem function and the delivery of ecosystem services. Quantifying the extent to which plant and soil biodiversity and function changes synchronously with forest restoration, and how these relationships change across contrasting climates and forest types globally is integral to solving major challenges for biodiversity conservation and climate change mitigation, and for predicting the future of forest rewilding under changing global climates.

Biodiversity and ecosystem function are often intimately positively linked across space and time_,_^[^ ^11^, ^14^, ^15^, ^16^, ^17^, ^18^ ^]^ yet it is still largely unknown whether these relationships are maintained after long‐term forest restoration, and across contrasting ecosystems. This knowledge gap has hampered our ability to identify potential biodiversity‐function tradeoffs during restoration. Most studies of changes in biodiversity and multiple function have focused at local or regional scales,^[^ ^13^, ^19^ ^]^ with very few investigating changes in both above and belowground biodiversity, and function, during forest restoration at global scales, or across contracting climates and forest types. Climate change is known to affect multiple ecosystems functions such as carbon sequestration and soil organic matter (SOM) decomposition.^[^ ^20^, ^21^ ^]^ Yet, much less is known about how climate regulates the simultaneous changes in biodiversity and function during forest restoration. Similarly, little is known about how contrasting key functional groups of forest biota such as ectomycorrhizal‐dominant compared with arbuscular mycorrhizal‐dominant forests^[^ ^22^, ^23^ ^]^ influence biodiversity and function during forest restoration. A rigorous assessment of the impacts of forest restoration on the multidimensionality of biodiversity and function across contrasting climates and forest types would provide information that is needed to support global initiatives to restore large area of Earth that formerly supported extensive forest systems.

Herein, we synthesized available data to investigate the fate of multidimensional forest rewilding under changing climates. The dataset comprised 120 studies, across five continents and spanning multiple contrasting ecosystem types and climate scenarios (e.g., dryland cf. mesic, angiosperms cf. conifers, evergreen cf. deciduous cf. mixed forests, arid cf. cold cf. tropical/temperate, arbuscular cf. ectomycorrhizal trees). Our synthesis included important information on tree and microbial biodiversity and five essential ecosystem services (i.e., plant biomass, microbial habitat, soil carbon, soil fertility, and SOM decomposition) associated with multiple functions (Table S1, Supporting Information). We investigated the changes in the restoration of these seven ecosystem attributes using response ratios (LnRR) calculated for available ecosystem properties across initial (*t*_0_) and older (*t*_x_) forest stand ages. Similar calculations were made for comparison with tree stand age. The response ratios of seven biodiversity and ecosystem function attributes (Table S1, Supporting Information) to forest restoration related to multiple ecosystem function were normally distributed (Figure S3, Supporting Information).

Using this forest restoration synthesis, we address the following key questions, aiming to better understand the consequences of large‐scale long‐term forest restoration processes: 1) Can forest restoration promote long‐term multiple dimensions of biodiversity and ecosystem function in forests? 2) To what extent are multi‐attributes interrelated, and can we identify a set of core attributes that act as simple proxies of multidimensional recovery? 3) Are there any tradeoffs between biodiversity and function during forest restoration? 4) How do differences in climate and forest type regulate the magnitude of change in soil biodiversity and function during forest restoration?

## 2 Results

**Figure 1:**
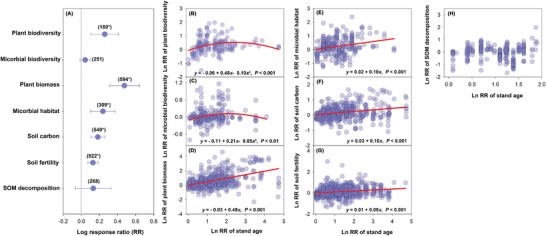
Global effect of forest restoration on multiple ecosystem attributes. A) Estimates (±95% CI) of the log response ratio for plant biodiversity, microbial biodiversity, plant biomass, microbial habitat, soil carbon, soil fertility and SOM decomposition. The vertical line was drawn at LnRR = 0. Number values for each bar indicate the sample size. The error bars indicated the 95% confidence interval (CI). If the CI did not overlap with zero, a response was considered to be significant. Relationships between response ratio of stand age with the response ratios (LnRR) of B) plant biodiversity, C) microbial biodiversity, D) plant biomass, E) microbial habitat, F) soil carbon, G) soil fertility, and H) SOM decomposition with forest restoration. Significant trends (*p* < 0.05) are shown with solid regression lines (e.g., relationship between stand age and forest restoration impacts (LnRR) on ecosystem function and biodiversity).

**Figure 2:**
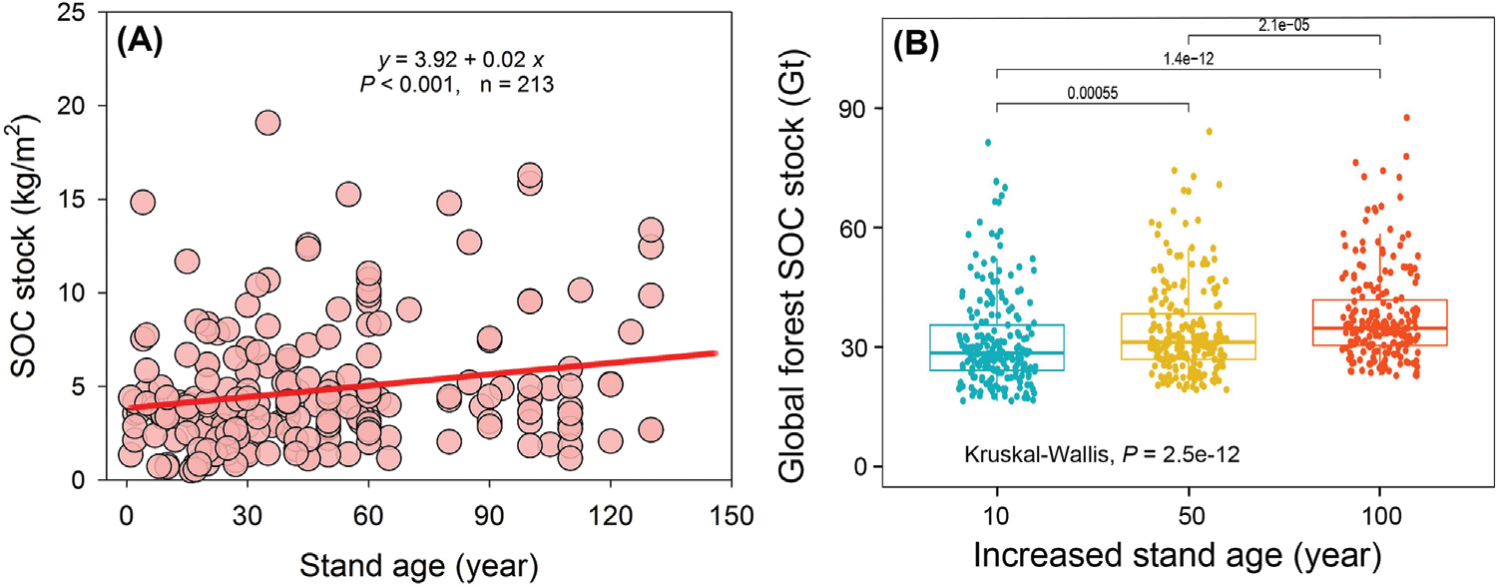
Potential effect of forest restoration on global soil organic carbon stock of The Bonn Challenge. A) Global relationships between SOC stock and stand age. B) Predicted global forest SOC stocks of The Bonn Challenge under future 10, 50, and 100 years. Kruskal–Wallis test was performed to examine the significant difference in SOC stocks between three stand age levels.

Our global synthesis provides solid evidence that, apart from SOM decomposition, multiple dimensions of ecosystem function, using the response ratio (LnRR as described in the Experimental Section), increased with forest restoration (**Figure** **1**A). Among the variables, plant biomass showed the largest increase in response to increasing stand age. Similarly, forest restoration had contrasting overall effects on biodiversity, with significant overall positive effects on plant biodiversity but no effects on soil biodiversity (Figure 1A). In addition, based on the global positive relationship between stand age and soil organic carbon (SOC) stock, we estimated that planting activities under The Bonn Challenge would increase soil C stock by 0.07 Gt C per year in the top 20 cm of soil by 2030. Planting 350 million hectares of forest would sequester 31.51, 34.32, and 37.84 Gt of carbon 10, 50, and 100 years, respectively, following forest restoration (**Figure** **2**).

**Figure 3:**
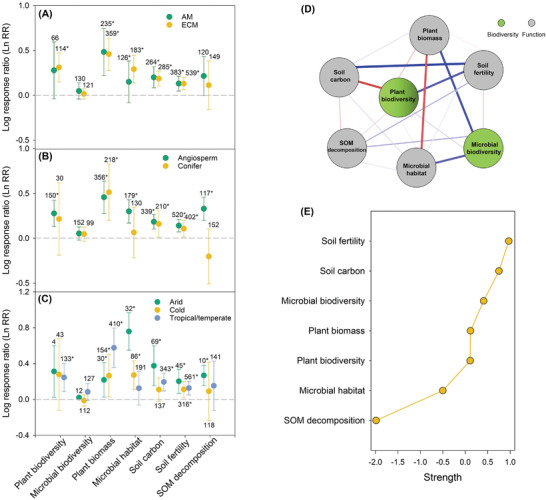
Effect of forest restoration on multiple ecosystem attributes. Estimates (±95% CI) of the log response ratio for plant biodiversity, microbial biodiversity, plant biomass, microbial habitat, soil carbon, soil fertility, and SOM decomposition during forest restoration under different A) mycorrhizal type, B) tree type, and C) Koppen climate type. The vertical line was drawn at LnRR = 0. Number values for each bar indicate the sample size. The error bars indicated the 95% confidence interval (CI). If the CI did not overlap with zero, a response was considered to be significant. D) Ecological connectivity network among eight ecosystems attributes in respond to forest restoration. Each attribute in the network is a node and connections represent partial correlation coefficients between two variables after conditioning on all other variables. The links with blue and red color indicate positive and negative correlation, respectively. The thickness of the links indicates the partial correlation coefficient. E) The strength of centrality indices of the network.

**Figure 4:**
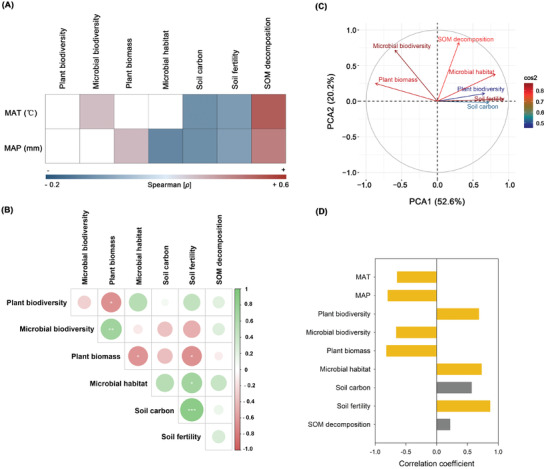
Links between multiple ecosystem attributes and climatic factors in respond to forest restoration. A) Significant correlations (Spearman; *p* < 0.05) between the temporal changes of multiple ecosystem attributes (lnRR) and climatic factor. B) The tradeoffs in the restoration (lnRR) among multiple ecosystem attributes. The circles' size presents correlation coefficient. The green and red color represents positive and negative correlation, respectively. Asterisk was considered to be significant. *, *p* < 0.05; **, *p* < 0.01; ***, *p* < 0.001. C) Principle component analysis (PCA) of the restoration (lnRR) of multiple ecosystem attributes. D) Bar plots of the spearman correlations coefficient between the restoration (lnRR) of multiple ecosystem attributes with principal component 1. Orange bars represent the correlations between ecosystems attributes and PCA1 that is considered significant (*p* < 0.05).

Importantly, our results further found that climate largely regulated the positive effects of forest rewilding on multiple dimensions of biodiversity and function, suggesting important climatic constrains in the multidimensional rewilding of global forests. Thus, although positive effects of forest restoration on most multiple ecosystem function were consistent across climates and forest types (**Figure** **3** and Figure S4, Supporting Information), the magnitude of these relationships varied across different environments. For example, we found that the average change in plant biomass with restoration was lower in drylands than mesic environments, but trends were the opposite for plant biodiversity, microbial habitat, and soil fertility. Similar results were found when comparing different climates (arid cf. cold cf. tropical/temperate) and forest types (angiosperm cf. conifers, arbuscular mycorrhizal cf. ectomycorrizal fungi). Network analyses demonstrated that recovery of attributes occurred in parallel, with the greatest expected influence for soil fertility, followed by soil carbon, microbial biodiversity, and plant biomass (Figure 3). This was also confirmed with principal components analysis (PCA) (**Figure** **4**C and Table S4, Supporting Information), showing similar associations among recovery of different forest attributes. Plant biodiversity was positively correlated with soil fertility but negatively correlated with soil carbon. Further, plant biomass could be negatively affected by microbial habitat but positively increased by microbial biodiversity.

Supporting these results, we found that changes in temperature and precipitation influenced the positive effects of forest restoration (LnRR) on multiple ecosystems function (Figure 4). Changes in SOM decomposition and microbial biodiversity were positively correlated with mean annual temperature (MAT), while negative correlations were found for soil carbon and soil fertility. Further, mean annual precipitation (MAP) was positively correlated with plant biomass and SOM decomposition, but negatively correlated with microbial habitat, soil carbon, and soil fertility. Overall, both MAT and MAP contributed with negative loadings on PCA1, which could explain 52.6% of the variation in multidimensional attributes (Figure 4D). In addition, forest restoration effects on multiple ecosystem function were also influenced by the particular climate scenario (Figures S5 and S6, Supporting Information).

Finally, we detected some undescribed important tradeoffs between biodiversity and ecosystem function in old forest restorations. Our results further revealed that while the association between stand age and restoration of ecosystem function is linear, the biodiversity‐stand age relationship exhibited a hump‐shaped relationship. Consequently, we found the hitherto undocumented disconnect between biodiversity and ecosystem function in very old forests, which was further supported by the negative correlations between biodiversity and function. More specifically, we found that changes in stand age (LnRR) were positively correlated with changes in multiple ecosystem function during forest restoration (Figure 1B–H), and there was no significant relationship between SOM decomposition and stand age (Figure 1G). Both plant biodiversity and microbial diversity exhibited unimodal (humped‐back) patterns with stand age, suggesting important reductions in biodiversity in old (centennial) forests, and explaining the overall lack of effect of stand age on soil biodiversity when the entire dataset was analyzed together. These analyses showed that during early forest development, both biodiversity and function increase, and although biodiversity is lower in very old forests, they still have the capacity to support high levels of ecosystem function (Figures 1B–H and 2). These results indicated the existence of important tradeoffs in very old forests during long‐term succession. Thus, multiple ecosystem functions such as plant biomass, soil fertility, carbon content, and microbial habitat (Figure 4B) followed similar positive trends with forest development, thereby ensuring the positive effect of restoration on function.

## 3 Discussion

Understanding the importance of forest rewilding for sustaining multiple components of biodiversity and function is essential for ensuring the long‐term success of global restoration initiatives during the UN's Decade on Restoration.^[^ ^6^, ^12^, ^24^ ^]^ Here, we found that, on average, forest restoration can promote plant biodiversity, and multiple dimensions of key ecosystem functions ranging from soil fertility to carbon stocks. These positive effects were consistent across climatic regimes and forest types, but the magnitude of the effects depended on environmental conditions, being greatest under arid climates and within arbuscular mycorrhizal forests. However, we found that biodiversity and function can become uncoupled in very old forests, suggesting the need for active intervention (e.g., selective harvest) to ensure healthy functional systems in these old forests. These findings suggest that arbuscular mycorrhizal forests under arid climates might be the best candidates to capture carbon, and to support key function such as carbon sequestration, microbial habitat, and soil fertility.

In general, we show an important coupling between ecosystem function and forests age. Several recent studies have identified the effects of forest development on ecosystems functions.^[^ ^8^, ^25^ ^]^ In our study, we found that forest development consistently promoted carbon sequestration in plants and soils, which is consistent with typical old‐growth forest succession in China^[^ ^26^ ^]^ and Spain.^[^ ^27^ ^]^ Increased plant biomass during forest development and aging would stimulate more photosynthetically fixed C as well as litter inputs to the soil, supporting soil fertility, microbial biomass, and decomposition rates.^[^ ^28^, ^29^ ^]^ Moreover, our results indicate that the 350 million hectares of forest planted under The Bonn Challenge could sequester >30 Gt C in the top 20 cm of the soil over the next century. This demonstrates that planting forests is an important strategy to achieve global carbon neutral goals and mitigate climate change.

Despite the overall positive influence of forest restoration on biodiversity and ecosystem function, we also found that the magnitude of this influence is driven by climatic and vegetation types. Climate change and ecological factors have long been recognized as major drivers of multiple ecosystem function.^[^ ^21^, ^29^, ^30^ ^]^ Here, our global synthesis provides evidence that rainfall and temperature can moderate the positive effects of forest restoration on multiple ecosystem function. Consequently, the effects of forest plantings on biodiversity and ecosystem function could wane as Earth becomes hotter and drier.^[^ ^31^, ^32^ ^]^ For example, we found that the average value of plant biomass under restoration was lower in drylands than mesic systems, and this was confirmed by the positive correlations between MAP and changes in plant biomass. Moreover, increases in soil carbon sequestration in forest plantations were negatively influenced by MAT. Thus, warmer ecosystems tend to accumulate less carbon as forests age. Greater microbial activity in warmer regions can usually accelerate the decay of SOM and increase C turnover rate. Thus, we found a negative correlation between MAT and changes in soil C during forest development.^[^ ^28^, ^33^ ^]^ In general, our results were consistent when conducting additional partial correlations between climatic variables and restoration influence on multiple ecosystem properties after controlling for changes in stand age (Figure S7, Supporting Information). This analysis further revealed that multiple dimensions of ecosystem restoration are more vulnerable to changes in rainfall than in temperature patterns with 86% of all restoration's ecosystem dimensions evaluated here being influenced by rainfall patterns (Figure S7, Supporting Information). These results suggest that as the planet becomes warmer and drier, the positive effects of forests on ecosystem functions will decline. This knowledge is important as a baseline to understand the potential influence of climatic changes in restoration efforts. Even so, we stress that our results based on historical conditions may be influenced by future changes in atmospheric CO_2_ levels, which could support more efficient forests in a drier world, and on the status of aquifers, which are rapidly being depleted by a growing global population.^[^ ^34^ ^]^ Scientists, managers, and policy makers should be aware of such tradeoffs so that they can predict the future of forests restoration.

Our results further reveal that forest type can influence the temporal changes in biodiversity and ecosystem function. Our results indicate that arbuscular mycorrhizal (AM) forests could capture more soil C than ectomycorrhizal (ECM) forests. AM trees tend to produce more easily decomposable litter, which promotes the formation of stable, mineral‐associated organic matter and results in a greater fraction of SOC than ECM trees.^[^ ^22^, ^28^, ^35^ ^]^ Further, a small group of ECM such as *Cortinarius acutus sensu lato* may exert a major influence by stimulating organic matter decomposition and thereby lowering soil carbon storage in forest ecosystems.^[^ ^36^ ^]^ Our study further suggests that angiosperms sequester more soil and plant carbon over time than conifers during forest development. Angiosperms have a greater photosynthetic capacity, water use efficiency, and specific leaf area, and a larger ratio between foliage area and plant biomass. This could markedly increase their resource utilization efficiency (e.g., light, nutrients) and stimulate their relative growth rate and thus, plant carbon storage.^[^ ^37^, ^38^, ^39^ ^]^ Angiosperms would be expected to enhance litter incorporation and stimulate accumulation of stable carbon stocks in soils owing to their greater N content and more favorable conditions for earthworms.^[^ ^40^, ^41^, ^42^ ^]^ Thus, our results highlight the fact that restoration with AM angiosperms would promote greater accumulation of C stocks and therefore be more effective against changing climates. Our findings suggest that planting AM angiosperm forests could result in greater ecosystem function in a shorter period of time.

Despite the generally positive influence of restoration on biodiversity and function, our work provides solid evidence that biodiversity becomes decoupled from ecosystem function as forests age. Reductions in plant biodiversity with forest age were offset by increases in multiple ecosystem functions such as plant biomass and soil C, suggesting a significant biodiversity‐function tradeoff in centennial forests. Competition‐to‐exclusion could explain this result in very old forests, whereby limiting resources (e.g., light) or abiotic stress (e.g., acidic soils reduce the diversity of bacteria)^[^ ^43^ ^]^ might simplify plant and soil biodiversity but continue to sustain high levels of plant and soil biomass under mature forest conditions.^[^ ^44^ ^]^ These results suggest that management might be needed in these old forests to maintained high levels of biodiversity and ecosystem function. Management actions could include controlled disturbances such as tree thinning, midstorey plantings, or cool season burning to reduce the biomass of dominant species (e.g., tree stands).^[^ ^45^ ^]^ Consistent with predictions under the intermediate disturbance hypothesis,^[^ ^46^ ^]^ this would likely increase biodiversity by releasing subordinate species from competitive exclusion.

In summary, forest restoration has been a core feature of global climate mitigation and biodiversity conservation strategies.^[^ ^8^ ^]^ Our meta‐analysis provides compelling evidence that forest development following restoration could promote ecosystem multidimensionality for above‐ and belowground biodiversity and multiple ecosystem functions, suggesting that, in general, the positive direction of these effects is consistent across climates and forest types. However, our results also indicate that the success of these restoration programs relies heavily on changing climates and forest type, which can retard any positive effect of rewilding on forest multidimensionality under climate change. Moreover, our study has identified for the first time, important tradeoffs in biodiversity and function in centennial forests, suggesting that active management might be necessary to support biodiversity of old forest stands. Our study provides new insights on the impacts of climate in the restoration of forest ecosystems based on the current literature. Yet, future work should aim to continue providing new insights and monitoring the restoration of these ecosystems, especially for those ecosystem properties (e.g., SOM decomposition) and environments (e.g., tropical forest) for which less information is available. Most contemporary Earth systems models still consider the synchronous positive change in biodiversity and ecosystem function or fail to account for differences in forest type.^[^ ^3^, ^7^ ^]^ Our studies suggest that next generation Earth systems models should explicitly incorporate functional trade‐offs in ecosystem restoration to improve predictions of how forest restoration affects ecosystem outcomes. Such information is vital if we are to fully appreciate the ecological consequences of forest restoration for sustaining biodiverse, functional systems on Earth, and their capacity to support vibrant human cultures.

## 4 Experimental Section

### Data Compilation

Journal articles were searched using Web of Science, Google Scholar, and the China National Knowledge Infrastructure database (CNKI) related to biodiversity and ecosystem function under forest restoration with the following key words combinations: “forest restoration” OR “secondary succession” OR “forest succession” OR “natural regeneration” OR “tree plantations” AND “soil carbon” OR “soil nitrogen” OR “soil phosphorus” OR “plant biomass” or “microbial communities” OR “microbial biomass” OR “diversity” OR “richness” OR “Shannon” OR “OTU.” Ecosystem function and biodiversity were partitioned into plant biodiversity, microbial biodiversity, plant biomass, microbial habitat, soil carbon, soil fertility, and SOM decomposition (Table S1, Supporting Information). Each attribute was only included in one category. To avoid bias in publication selection, the following criteria are conducted to select the studies compiled in their database: i) All forests were subjected to an original disturbance (e.g., harvesting) and are now on an on‐going secondary successional trajectory; ii) the restoration age on all stages should be clearly reported; iii) at least one of the selected variables as well as at least two restoration stages in forest restoration experiment was recorded; iv) restoration efforts should be less than 150 years; v) all restoration stage should be observed in the same region with same climate conditions; vi) the means of selected variables in all stages could be extracted directly from tables, digitized graphs, or contexts. Using the six above selection criteria, 120 papers (Table S1, Text S1 and PRISMA diagram in Figures S1 and S2, Supporting Information) were selected to evaluate how forest restoration influence biodiversity and function globally. Among these studies, 21 addressed plant biodiversity, 28 addressed microbial biodiversity, 58 addressed plant biomass, 29 addressed microbial habitat, 100 addressed soil carbon, 18 addressed SOM decomposition, and 92 addressed soil fertility.

Data were also collected for soil properties including pH, bulk density, soil moisture, and clay content. Mean values were taken from tables or extracted from figures using GetData software (version 2.22). To avoid climate bias, MAP and MAT were extracted from the WorldClim database (www.worldclim.org/data/bioclim.html) using the geographical coordinates of the study sites. For each site in the dataset, the aridity index (AI) was calculated as the ratio of annual precipitation over potential evaporation; the latter term was obtained from the WorldClim database. Climate biomes were classified as dryland with aridity index < 0.65, and mesic with aridity index > 0.65. Koppen classification was divided into arid, cold, and tropical‐temperate zones using the method from Beck et al.^[^ ^47^ ^]^ The information was tabulated on the mycorrhizal association of the dominant species as AM (arbuscular mycorrhizal fungi) and ECM (ectomycorrizal fungi) at each experimental site, using the database of Wang and Qiu^[^ ^48^ ^]^ and Soudzilovskaia et al. (2020).^[^ ^49^ ^]^ Most analyses in this study were focused on AM and ECM, as biomes with nonmycorrhizal fungi (NMF) were too few for allowing to conduct any analyses on them. Tree species were also divided into different functional groups (angiosperms and conifers), leaf life span (evergreen, deciduous, and mixed forests) using information available in the literature to test whether the restoration effects could be affected by tree functional type.

### Data Analysis

The methods of Mooney et al.)^[^ ^50^ ^]^ and Crouzeilles et al.^[^ ^9^ ^]^ were followed to calculate the log response ratio (LnRR) of all ecosystem variables for all age combinations during forest development. The effect size was calculated as the natural logarithm (Ln) of the response ratio (RR) as LnRR = Ln (*t_x_*/*t*_0_), where *t_x_* is the mean value of a particular variable within an older forest stand and *t*_0_ is the value for the youngest forest age. For example, if one study reported soil carbon levels within young, moderate, and old stands, a log response ratio was able to calculate for total soil carbon for two stand age combinations, i.e., young versus old, young versus moderate and moderate versus old. The log response ratio was negative when the value of a particular variable was lower in the older stand. The average LnRR values for all variables within a given category (e.g., plant biomass) (Table S1, Supporting Information) were then calculated. Examination of funnel plots of effect sizes versus sample size did not suggest any publication biases that would be expected in cases of underreporting of nonsignificant results due to low replication.^[^ ^51^ ^]^ To maintain consistency with previous meta‐analysis, the conservative approach was taken of not weighting effect sizes by their variance.^[^ ^50^, ^52^, ^53^ ^]^ Currently, nearly half of published meta‐analyses have used the response ratio metric to explore the treatments effects on concerned variables.^[^ ^9^, ^54^ ^]^ This approach had an advantage over other weighted metrics because it required only means data for the dependent variables for two groups, while other weighted metrics required some measure of variance (e.g., SD) and sample sizes for both values in each pair.^[^ ^55^ ^]^ This could solve the potential problem where many studies did not provide a measure of variance, SD or sample size, and allowed to utilize a larger number of potential studies on which to perform these analyses.^[^ ^9^ ^]^

### Statistical Analysis

The methods used by Mooney et al.^[^ ^50^ ^]^ and Crouzeilles et al.^[^ ^9^ ^]^ were followed to quantify the average effects of forest restoration on plant biodiversity, microbial biodiversity, plant biomass, microbial habitat, soil carbon, soil fertility, and SOM decomposition using linear mixed model in R package *lme 4* with LnRR as the dependent variable. The significance of these models was tested with likelihood ratio tests. Estimates of LnRR were derived from restricted maximum likelihood and 95% confidence intervals for the estimates obtained from the likelihood profile. The spatiotemporal variations of ecosystems function (e.g., plant biomass) and biodiversity (e.g., plant biodiversity) were examined with LnRR of stand age, MAT and MAP.

To visualize the functional tradeoffs in ecosystem restoration, a correlations matrix was constructed using the R package “corrplot.” Correlation coefficients and significance (*p* < 0.05) were presented as a heat map with contrasting colors representing positive and negative correlations and circle size depicting the magnitude of the coefficient. The probability density distributions of all attributes using “ggridges” in the R package were then fitted. This method could classify ridge regression predictors in disjoint groups of conditionally correlated variables and derived different penalties (i.e., shrinkage parameters) for predictors groups.^[^ ^56^ ^]^ It had the advantage of combining the ridge regression method with graphical model for ill‐conditioned data or high‐dimensional data.

Based on the global relationships between stand age and SOC stock, soil C stocks in the uppermost 20 cm of the profile to predict potential global forest SOC stocks under The Bonn Challenge (250 and 350 million hectares of forest by 2020 and 2030, respectively) were estimated. Soil bulk density values of 1 Mg m^−3^ under 10, 50, and 100 year scenarios were used. Kruskal–Wallis test was performed to examine the significant difference in global forest SOC stock between three forest stand age under The Bonn Challenge.

Network analysis was used to evaluate how multiple forest attributes were associated, and to assess which attribute was most central among these attributes, and to identify potential clusters of closely linked attributes. Specifically, a pairwise Markov random field network model to estimate the partial correlation networks, which was optimized using the extended Bayesian information criterion (“EBICglasso”) with the “bootnet” package.^[^ ^57^ ^]^ The centrality was computed by summing the absolute values of partial correlations to obtain the higher values quantified as important attributes to assess the importance of attributes in the network.^[^ ^57^ ^]^ Strength was calculated from accumulated values of absolute partial coefficients between a focal attribute and all other connected attribute in the network. Strength was standardized by subtracting the mean from the specific values and dividing it by the standard deviation. Large strength values indicated high central attributes. More details about the Network analysis process could be found from Poorter et al.^[^ ^12^ ^]^

Considering the nonlinear methods might over‐parameterized on diverse data, the PCA was conducted on the multivariate space of the forest ecosystems attributes as described by Migliavacca et al.^[^ ^58^ ^]^ Specifically, multiecosystems attributes were standardized using Z‐transformation. The explained variance of each components (PCAs), as well as the loadings of each ecosystem attributes, demonstrating the contributions of each ecosystem function to the concerned PCAs were then extracted. Relationships between PCAs with each ecosystem attributes as well as climatic factors (i.e., MAT and MAP) were explored by spearman correlation analysis. In addition, partial correlation analyses was conducted in SPSS to cross‐validate the influence of climate (MAT and MAP) on LnRR indexes controlling for LnRR stand age.

## Conflict of Interest

The authors declare no conflict of interest.

## Author Contributions

M.D.B. designed the study. G.Y.Z. collected the data and analyzed data. G.Y.Z., X.H.Z., and M.D.B. wrote the first draft of the manuscript with significant contributions of D.J.E. Y.J.S., R.Q.L., L.Y.Z., Y.H.H., and Z.G.D. contributed to the writing of the paper.

## Supporting information

Supporting Information

## Data Availability

The data that support the findings of this study are available from the corresponding author upon reasonable request.
