# Supplementary material for: Temperature and Rainfall Patterns Constrain the Multidimensional Rewilding of Global Forests
Source: Adv Sci (Weinh). 2022 Apr 25;9(18):2201144. doi: 10.1002/advs.202201144 (PMC9218649; doi:10.1002/advs.202201144)
Supplement: Supplementary file 1 — Supporting Information [file ADVS-9-2201144-s001.pdf]

ADVANCED SCIENCE
Open Access

Supporting Information

for *Adv. Sci.*, DOI 10.1002/advs.202201144

Temperature and Rainfall Patterns Constrain the Multidimensional Rewilding of Global Forests

Guiyao Zhou, Xuhui Zhou, David J. Eldridge, Ximei Han, Yanjun Song, Ruiqiang Liu, Lingyan Zhou, Yanghui He, Zhenggang Du and Manuel Delgado-Baquerizo**

Supporting information

Temperature and rainfall patterns constrain the multidimensional rewilding of global forests

Content:

Table S1. Attributes used to derive the seven ecosystem functions and biodiversity.

Table S2. The Reporting Standards for Systematic Evidence Syntheses in Environmental Research (ROSES) report for our meta-analysis.

Table S3. Literature included in our dataset for the meta-analysis.

Table S4. Contributions of multiple ecosystem attributes to PCA 1 of the principal component analysis.

Figure S1. Article selection process using Preferred Reporting Items for Systematic Reviews (PRISMA) guidelines.

Figure S2. Global distribution of the selected stand forest. The blue and red circles indicate which experiment provided selected angiosperm and conifers stands, respectively. Based green map represents the distribution of global evergreen, deciduous and mixed forests. The observations of Angiosperm and conifers forests were 141 and 65, respectively.

Figure S3. Probability density of the forest restoration effect on plant biodiversity, microbial biodiversity, plant biomass, microbial habitat, soil carbon, SOM decomposition and soil fertility. Results are based on 21 studies for plant biodiversity, 28 studies for microbial biodiversity, 58 studies for plant biomass, 29 studies for microbial habitat, 100 studies for soil carbon, 18 studies for SOM decomposition and 92 studies for soil fertility.

Figure S4. Estimates (±95% CI) of the log response ratio for plant biodiversity, microbial biodiversity, plant biomass, microbial habitat, soil carbon, soil fertility and SOM decomposition of different climate type (A, dryland and mesic) and tree functional type (B, evergreen, deciduous and mixed forests). The vertical line was drawn at LnRR=0. Number values for each bar indicate the sample size. The error bars indicated the 95% confidence interval (CI). If the CI did not overlap with zero, a response was considered to be significant.

Figure S5. Relationships between stand age with the response ratios (LnRR) of plant biodiversity (A), microbial biodiversity (B), plant biomass (C), microbial habitat (D), soil carbon (E), soil fertility (F) and SOM decomposition (G) for

dryland and mesic forests.

Figure S6. Relationships between stand age with the response ratios (LnRR) of plant biodiversity (A), microbial biodiversity (B), plant biomass (C), microbial habitat (D), soil carbon (E), soil fertility (F) and SOM decomposition (G) for arid, cold, and tropical/temperate forests.

Figure S7 Partial correlation analyses reveals that climate is significantly correlated with the restoration of multiple ecosystem attributes (lnRR) even when controlling for changes across different age ranges (lnRR stand age).

Text S1. The reference lists of selected literature.

Table S1 Attributes used to derive the seven ecosystem functions and biodiversity.

Ecosystem functions & biodiversity	Attribute
Plant biomass	Basal area, coverage, DBH, stand volume, community height, aboveground biomass, belowground biomass, total biomass, litter biomass, forest floor biomass and dead woody biomass
Plant biodiversity	Richness, evenness, Simpson and Shannon index
Soil carbon	Soil total carbon stock, soil organic carbon content, soil organic carbon content stocks, soil organic matter content
Soil fertility	Total nitrogen content, total nitrogen stocks, ammonium nitrogen concentration, nitrate nitrogen concentration, mineral N concentration, total phosphorus concentrations, available phosphorus concentrations, and total potassium concentrations
SOM decomposition	N mineralization, urease, cellulolytic enzymes, invertase, phosphatase, catalase, polyphenol oxidase, peroxidase, β-d-cellubiosidase, xylosidase and cellulase dehydrogenase, β-Glucosidase, N-Acetyl-β-D-glucosaminidase, α-glucosidase, β-D-xylanase, Leucine-α-aminopeptidase
Microbial habitat	Total PLFAs, bacterial biomass, fungi biomass, Gram-positive bacteria, Gram-negative bacteria, Arbuscular mycorrhizal fungi biomass, Actinomycetes biomass, microbial biomass carbon, microbial biomass nitrogen, microbial biomass phosphorus
Microbial biodiversity	Bacterial richness, bacterial evenness, bacterial Simpson, bacterial Shannon, bacterial Chao 1, bacterial OTU, fungi richness, fungi evenness, fungi Simpson, fungi Shannon, fungi Chao 1, fungi OTU, microbial richness, microbial evenness, microbial Simpson, and microbial Shannon

Table S2 The Reporting Standards for Systematic Evidence Syntheses in Environmental Research (ROSES) report for our meta-analysis.

Section / sub-section	Topic	Description	Further explanation	Checklist/Meta-data	Author response
**Title**	Title	The title must indicate that it is a systematic review protocol, and must indicate if it is an update/amendment: e.g. "A systematic review update protocol...".	The title should normally be the same or very similar to the review question.	Meta-data	Climate constrains the multidimensional rewilding of global forests
**Type of review**	Type of review	Select one of the following types of review: systematic review, systematic review update, systematic review amendment, systematic review from a systematic map	See CEE Guidance on amendments and updates	Meta-data	systematic review
**Authors contacts**	Authors contacts	The full names, institutional addresses, and email addresses for all authors must be provided.		Checklist	Yes
**Abstract**	Structured summary	Abstract must not exceed 350 words and must include two sections 1) Background, the context and purpose of the review, including the review question; 2) Methods, how the review will be conducted and the outputs that are expected (specifically mention search strategy, inclusion criteria, critical appraisal, data extraction and synthesis).		Checklist	Yes
**Background**	Background	Describe the rationale for the review in the context of what is already known. Protocol must indicate why this study was necessary and what it aims to contribute to the field.	A theory of change and/or conceptual model can be presented that links the intervention or exposure to the outcome.	Checklist	Yes

**Stakeholder engagement**	Stakeholder engagement	The planned/actual role of stakeholders throughout the review process (e.g. in the formulation of the question) must be described and explained (using a broad definition of 'stakeholder', including e.g. researchers, funders and other decision-makers.		Checklist	Yes
**Objective of the review**	Objective	Describe the primary question and secondary questions (when applicable).	The primary question is the main question of the review. Secondary questions are usually linked to sources of heterogeneity (effect modifiers).	Checklist	Yes
	Definitions of the question components	Break down and summarise question key elements e.g. population, intervention(s)/exposure(s), comparator(s), and outcome(s).		Meta-data	Yes
**Methods**					
**Searches**	Search strategy	Detail the planned search strategy to be used, including: database names accessed, institutional subscriptions (or date ranges subscribed for each database), search options (e.g. 'topic words' or 'full text' search facility), efforts to source grey literature, other sources of evidence (e.g. hand searching, calls for evidence/submission of evidence by stakeholders).	Details regarding search strategy testing should be provided.	Checklist	Yes

	Search string	Provide Boolean-style full search string and state the platform for which the string is formatted (e.g. Web of Science format)		Meta-data	[Web of science, Google Scholar, CNKI] ("forest restoration" OR "secondary succession" OR "forest succession" OR "natural regeneration" OR "tree plantations", AND "soil carbon" OR "soil nitrogen" OR "soil phosphorus" OR "plant biomass" or "microbial communities" OR "microbial biomass" OR "diversity" OR "richness" OR "Shannon" OR "OTU")
	Languages – bibliographic databases	List languages to be used in bibliographic database searches.		Meta-data	English
	Languages – grey literature	List languages to be used in organizational websites searches and web-based search engines.		Meta-data	English and Chinese
	Bibliographic databases	Provide the number of bibliographic databases to be searched.		Meta-data	n/a
	Web – based search engines	Provide the number of web – based search engines to be searched.		Meta-data	Google Scholar
	Organisational websites	Provide the number of organisational websites to be searched.		Meta-data	n/a
	Estimating the comprehensiveness of the search	Describe the process by which the comprehensiveness of the search strategy was assessed (i.e. list of benchmark articles).		Checklist	Yes
	Search update	Describe any plans to update the searches during the conduct of the review.	Optional. A search update is good practice if original searches were performed more than two years prior	Checklist	Yes

			to review completion.		
**Article screening and study inclusion criteria**	Screening strategy	Describe the methodology for screening articles/studies for relevance/eligibility.		Checklist	Yes
	Consistency checking	Describe clearly the process for checking consistency of decisions including the levels at which consistency checking will be undertaken and estimated proportion of articles/studies that will be screened and checked for consistency by two or more reviewers (e.g. Titles (10%), abstracts (10%), full text (10%)).		Checklist	Yes
	Inclusion criteria	Describe the inclusion criteria used to assess relevance of identified articles/studies. These must be broken down into the question key elements (e.g. relevant subject(s), intervention(s)/exposure(s), comparator(s), outcomes, study design(s)) and any other restrictions (e.g. date ranges or languages).		Checklist	Yes
	Reasons for exclusion	State that you will provide a list of articles excluded at full text with reasons for exclusion.		Checklist	Yes
**Critical appraisal**	Critical appraisal strategy	Describe here the method you propose for critical appraisal of study validity (including assessment of individual studies and the evidence base as a whole).		Checklist	Yes

	Critical appraisal used in synthesis	Describe how the information from critical appraisal will be used in synthesis.		Checklist	Yes
	Consistency checking	Describe how repeatability of critical appraisal of study validity will be tested.		Checklist	Yes
**Data extraction**	Meta-data extraction and coding strategy	Describe the method for meta-data extraction and coding for studies (potentially providing forms/data sheets (ideally piloted), list if variables to be extracted as meta-data and those that will be coded).		Checklist	Yes
	Data extraction strategy	Describe the method for extraction of qualitative and/or quantitative study findings (potentially providing forms/data sheets (ideally piloted))		Checklist	Yes
	Approaches to missing data	Describe any processes for obtaining and confirming missing or unclear information or data from authors.		Checklist	Yes
	Consistency checking	Describe how repeatability of the meta-data/data extraction process will be tested.		Checklist	Yes
**Potential effect modifiers/reasons for heterogeneity**	Potential effect modifiers/reasons for heterogeneity	Provide a list of and justification for the effect modifiers /reasons for heterogeneity that will be considered in the review. Also provide details of how the list was compiled (including consultation of external experts).	The list should not be exhaustive but a short list of those variables thought to be most important and amenable to analysis.	Checklist	Yes
**Data synthesis and presentation**	Type of synthesis	State the type of synthesis conducted as part of the systematic review (narrative only, narrative and quantitative, narrative and qualitative, narrative,		Meta-data	narrative and mixed-methods

		qualitative and quantitative, narrative and mixed-methods)			
	Narrative synthesis strategy	Describe methods to be used for narratively synthesising the evidence base in the form of descriptive statistics, tables (including any map databases) and figures.	Vote-counting (tallying of studies based on the direction or significance of their findings) must be avoided. Must include a summary of the outputs of critical appraisal of the evidence base as a whole.	Checklist	Yes
	Quantitative synthesis strategy	If data are appropriate for quantitative synthesis, describe planned methods for calculating effect sizes, methods for handling complex data, statistical methods for combining data from individual studies, and any planned exploration of heterogeneity (e.g. sensitivity analysis, subgroup analysis and meta-regression). If all studies may not be selected for synthesis explain criteria for selection (e.g. incomplete or missing information).	Compulsory if appropriate for data	Checklist	Yes
	Qualitative synthesis strategy	Describe methods to be used for synthesising qualitative data and justify your methodological choice. Describe if and how you plan to analyse subgroups/subsets of data. If all studies may not be selected for synthesis explain criteria for selection (e.g. incomplete or missing information).	Compulsory if appropriate for data	Checklist	Yes

	Other synthesis strategies	Describe any other approaches to be used for synthesising data or combining qualitative and quantitative synthesis (e.g. mixed-methods) and justify your methodological choice.	Compulsory if appropriate for data	Checklist	n/a
	Assessment of risk of publication bias	Describe planned methods for examining the possible influence of publication bias on the synthesis.	For quantitative syntheses this may be done using diagnostic plots or statistical tests	Checklist	Yes
	Knowledge gap identification strategy	Describe the methods to be used to identify and/or prioritise key knowledge gaps (unrepresented or underrepresented subtopics that warrant further primary research).	Optional	Checklist	Yes
	Demonstrating procedural independence	Describe the role of systematic reviewers (who have also authored articles to be considered within the review) in decisions regarding inclusion or critical appraisal of their own work.	Reviewers who have authored articles to be considered within the review should be prevented from unduly influencing inclusion decisions, for example by delegating tasks appropriately.	Checklist	Yes
**Declarations**	Competing interests	Describe of any financial or non-financial competing interests that the review authors may have.		Checklist	Yes

Table S3 Literature included in our dataset for the meta-analysis.

References	Plant biodiversity	Microbial biodiversity	Plant biomass	Microbial habitat	Soil carbon	Soil fertility	SOM decomposition
Zethof et al., 2018					yes	yes	
Yankelevich et al., 2007			yes		yes	yes	
Winbourne et al., 2018			yes		yes		
Shi et al., 2012		yes		yes	yes	yes	
Zhao et al., 2020		yes	yes	yes	yes		
Shi et al., 2017		yes	yes	yes	yes	yes	
Lin et al., 2015	yes	yes		yes	yes	yes	
Liu et al., 2013		yes					
Wu et al., 2016		yes		yes	yes	yes	
Duo et al., 2012		yes	yes	yes	yes	yes	
Xiong et al., 2021					yes	yes	
Liu et al., 2012		yes					yes
Lu et al., 2017		yes	yes	yes	yes	yes	yes
Ren et al., 2018		yes		yes	yes	yes	
Chen et al., 2013			yes		yes	yes	
Tongkoom et al., 2018	yes		yes		yes	yes	
Yuan et al., 2019					yes	yes	yes
Wang et al., 2016				yes			
Zaitsev et al., 2012	yes						
Yan et al., 2008			yes		yes		
Shao et al., 2017			yes		yes	yes	
Liu et al., 2019		yes	yes	yes	yes	yes	
Jones et al., 2019			yes		yes	yes	
Turpeinen et al., 2020			yes		yes	yes	
Deng et al., 2013					yes	yes	
Zhang et al., 2018	yes		yes	yes	yes	yes	yes
Zhao et al., 2018		yes		yes	yes		
Zhang et al., 2018			yes		yes	yes	
Lu et al., 2019			yes	yes	yes	yes	
Zhao et al., 2015					yes		
Mo et al., 2013					yes	yes	
Dou et al., 2013			yes		yes	yes	
Hume et al., 2016			yes		yes	yes	
Powers et al., 2009					yes	yes	
Jia et al., 2005				yes	yes	yes	
Toniato et al., 2004					yes		
Cai et al., 2018		yes			yes	yes	
Guggenberger et al., 1999					yes	yes	
Zhu et al., 2010		yes		yes	yes	yes	
Liu et al., 2011			yes		yes	yes	
Amazonas et al., 2011						yes	

Martelletti et al., 2019						yes	
Wu et al., 2020				yes	yes	yes	yes
Wu et al., 2018					yes	yes	
Jin et al., 2019	yes	yes			yes	yes	yes
Yan et al., 2006			yes	yes		yes	
Yan et al ., 2009	yes				yes	yes	yes
Yang et al., 2020							
Zhang et al., 2016				yes	yes	yes	
Zeng et al., 2015				yes	yes	yes	
Zeng et al., 2013			yes		yes		
Yao et al., 2020			yes	yes			yes
Saynes et al., 2005			yes		yes	yes	yes
Wang et al., 2020			yes		yes		
He et al., 2016		yes			yes	yes	yes
Xu et al., 2018					yes	yes	
Chen et al., 2014			yes		yes	yes	
Feudis et al., 2020					yes	yes	
Wang et al., 2018	yes		yes				
Huang et al., 2015	yes		yes		yes	yes	
Aravena et al ., 2002	yes		yes		yes	yes	
Lucas-Borja et al., 2019			yes		yes	yes	
Brearley et al., 2011			yes		yes	yes	
Zhang et al., 2010			yes		yes	yes	
Castellanos-Castro et al., 2015			yes		yes	yes	
Lu et al., 2016	yes				yes	yes	
McClellan et al., 2018	yes		yes				
Cao et al., 2020			yes		yes	yes	
Alberti et al., 2008			yes		yes	yes	
Lu et al., 2018	yes		yes		yes	yes	
Badalamenti et al., 2019			yes		yes	yes	
Emmer et al., 1998			yes		yes	yes	
Bai et al., 2019		yes		yes	yes	yes	
Fang et al., 2010			yes		yes		
Kaneda et al ., 2020					yes	yes	
Smith et al., 2015							yes
Han et al., 2020						yes	
Li et al., 2015	yes	yes			yes	yes	
Han et al., 2015			yes		yes		
Bush et al., 2008					yes	yes	
Tang et al., 2010	yes		yes		yes	yes	
Cindy et al., 2014	yes		yes		yes	yes	
Valdespino et al., 2009			yes		yes	yes	yes
Hasselquist et al., 2010			yes				

Reyes et al., 2019		yes			yes		
Huang et al., 2013					yes	yes	yes
Yuan et al., 2012			yes				
Aidar et al., 2003					yes	yes	
Liu et al., 2002					yes	yes	
Li et al., 2013			yes	yes	yes	yes	
Dang et al., 2017		yes	yes		yes	yes	
Chen et al., 2012			yes	yes	yes	yes	
Woodbury et al., 2020					yes	yes	
Liu et al., 2019		yes			yes	yes	
Liu et al., 2020			yes				
LiuG et al., 2019		yes			yes	yes	
LiuY et al., 2020	yes	yes	yes	yes	yes	yes	
Luan et al., 2011			yes				
Bauters et al., 2019	yes		yes		yes		
Ostertag et al., 2008			yes		yes	yes	
Negrete-Yankelevich et al., 2008		yes			yes	yes	
Pereira et al., 2020		yes					
Pérez et al., 2004			yes				yes
Perakis et al., 2015					yes	yes	
Ma et al., 2020					yes	yes	
Shao et al., 2019		yes			yes	yes	
Schwendenmann et al., 2000					yes	yes	
Feng et al., 2020			yes	yes			
Yassir et al., 2015					yes	yes	
Song et al., 2015	yes				yes	yes	
Huang et al., 2018					yes	yes	
Sokołowska et al., 2020				yes	yes	yes	yes
Mylliemngap et al., 2016	yes		yes		yes	yes	yes
TangC et al., 2010					yes	yes	
Wang et al., 2017			yes		yes	yes	
Mo et al., 2006					yes	yes	
Yan et al., 2020	yes	yes		yes	yes	yes	
WangJ et al., 2020	yes	yes		yes	yes	yes	yes
WangH et al., 2020			yes		yes	yes	
YanW et al., 2020				yes	yes	yes	yes

Table S4 Contributions of multiple ecosystem attributes to PCA 1 of the principal component analysis.

	Eigen vectors	Contribution (%)	cos2
Plant biodiversity	0.35	11.97	0.44
Microbial biodiversity	-0.31	9.60	0.35
Plant biomass	-0.45	20.45	0.75
Microbial habitat	0.42	17.94	0.66
Soil carbon	0.38	14.17	0.52
Soil fertility	0.48	23.36	0.86
SOM decomposition	0.16	2.51	0.09

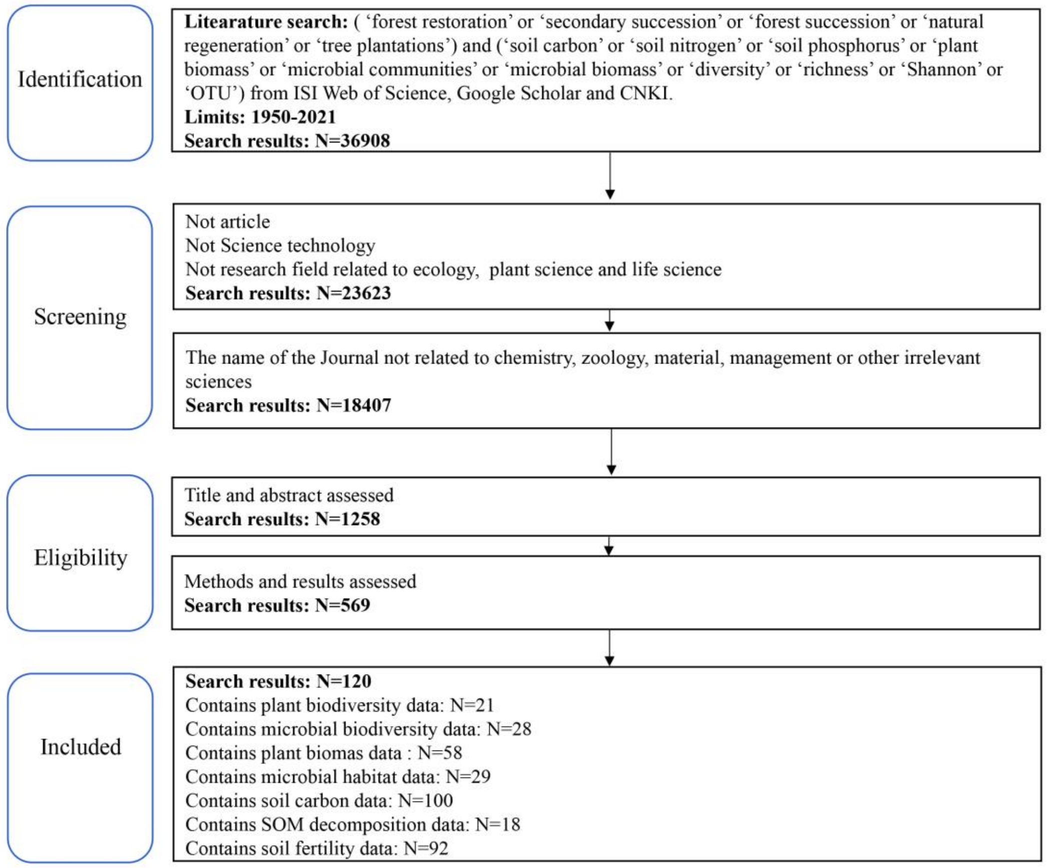

Figure S1 Article selection process using Preferred Reporting Items for Systematic Reviews (PRISMA) guidelines.

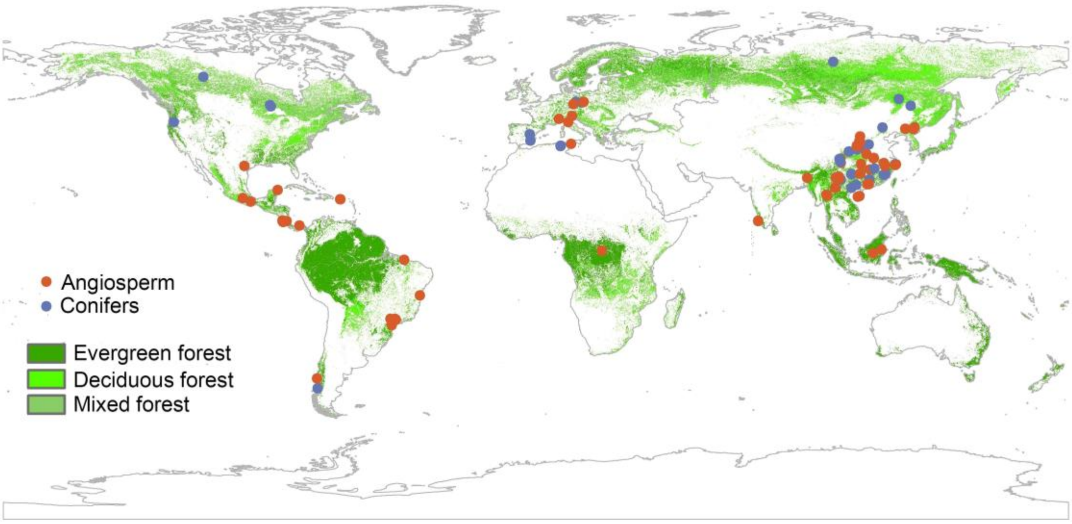

Figure S2 Global distribution of the selected stand forest. The blue and red circles indicate which experiment provided selected angiosperm and conifers stands, respectively. Based green map represents the distribution of global evergreen, deciduous and mixed forests. The observations of Angiosperm and conifers forests were 141 and 65, respectively.

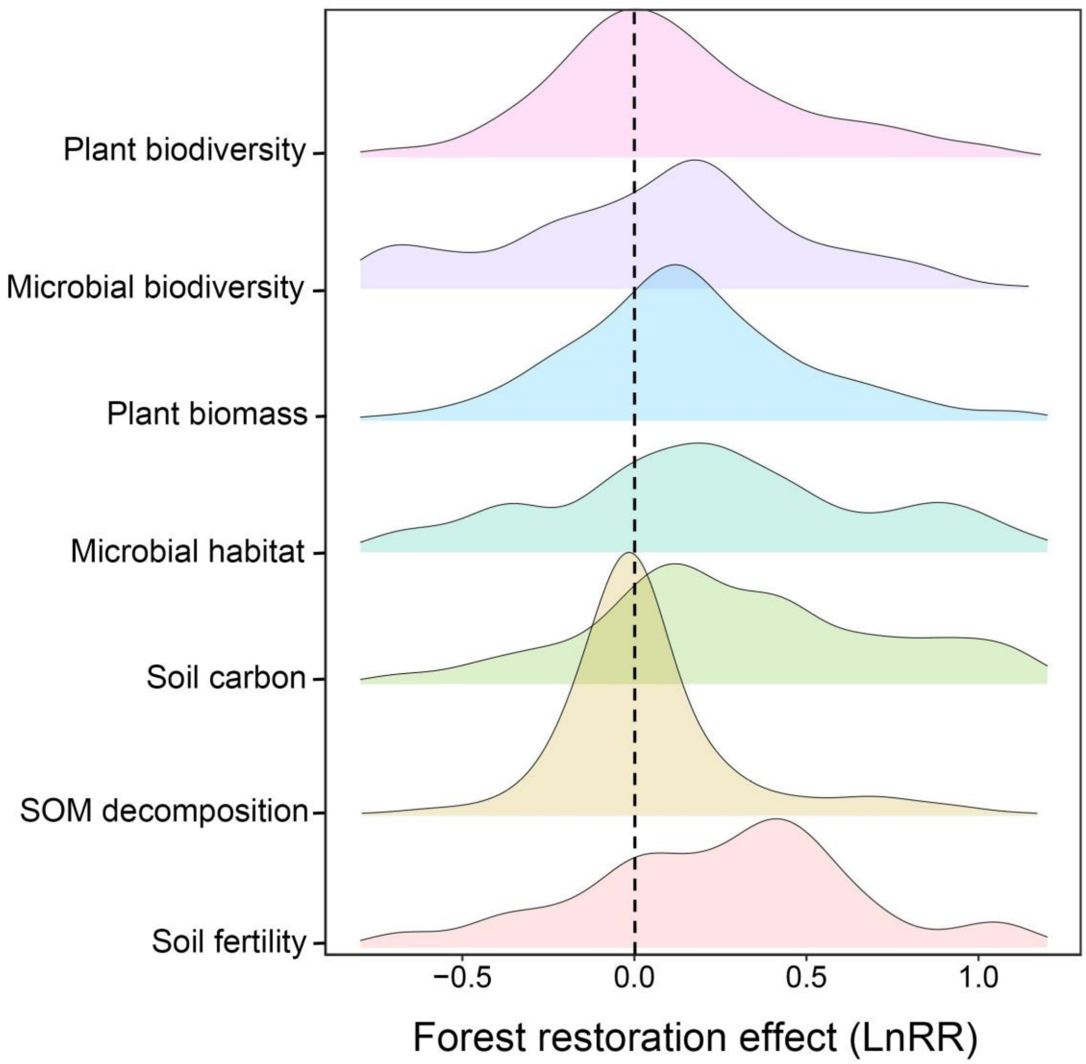

Figure S3 Probability density of the forest restoration effect on plant biodiversity, microbial biodiversity, plant biomass, microbial habitat, soil carbon, SOM decomposition and soil fertility. Results are based on 180 observations for plant biodiversity, 251 observations for microbial biodiversity, 594 observations for plant biomass, 309 observations for microbial habitat, 549 observations for soil carbon, 268 observations for SOM decomposition and 922 observations for soil fertility.

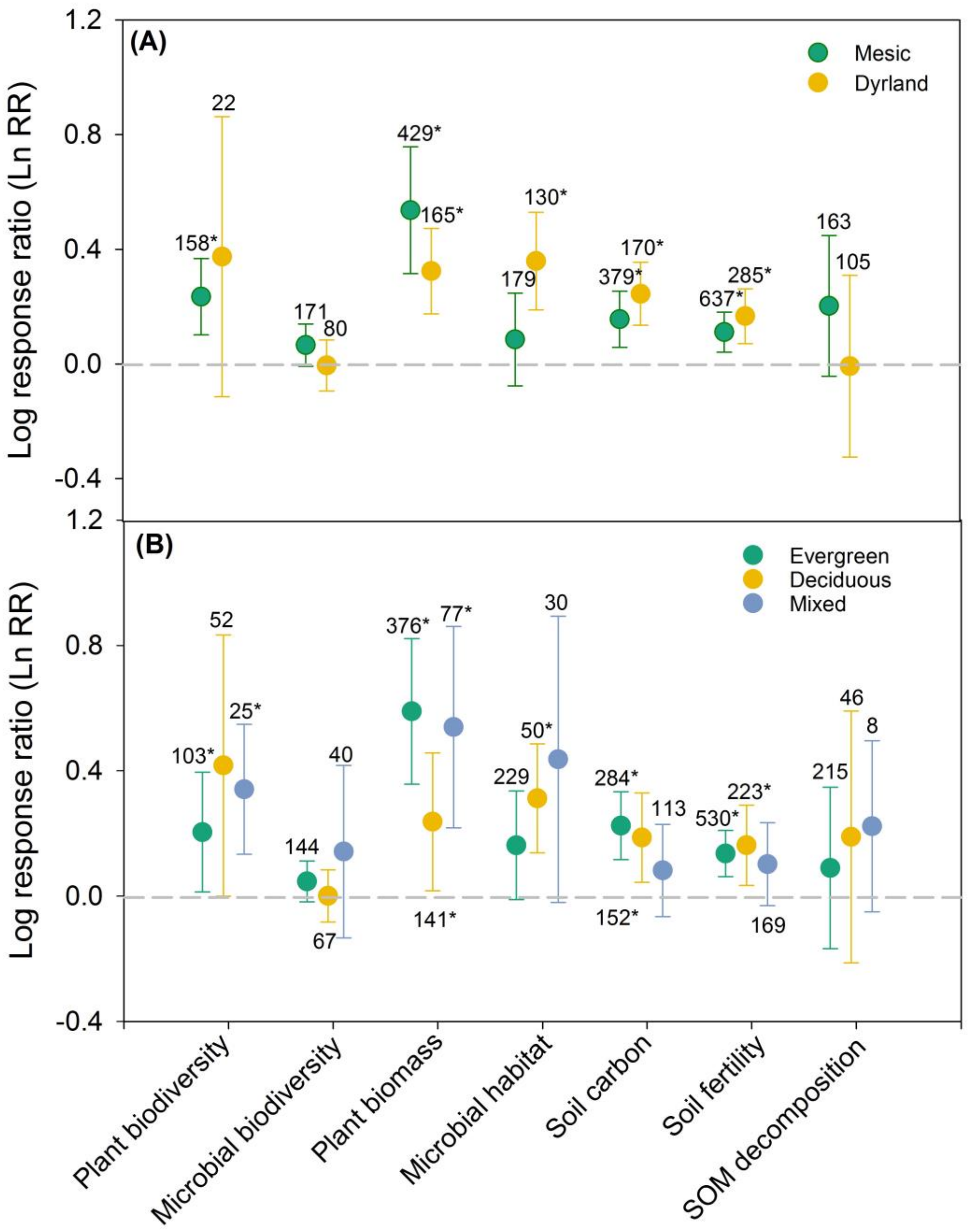

Figure S4 Estimates (±95% CI) of the log response ratio for plant biodiversity, microbial biodiversity, plant biomass, microbial habitat, soil carbon, SOM decomposition, and soil fertility of different climate type (A, dryland and mesic) and tree functional type (B, evergreen, deciduous and mixed forest). The vertical line was drawn at LnRR=0. Number values for each bar indicate the sample size. The error bars indicated the 95% confidence interval (CI). If the CI did not overlap with zero, a response was considered to be significant.

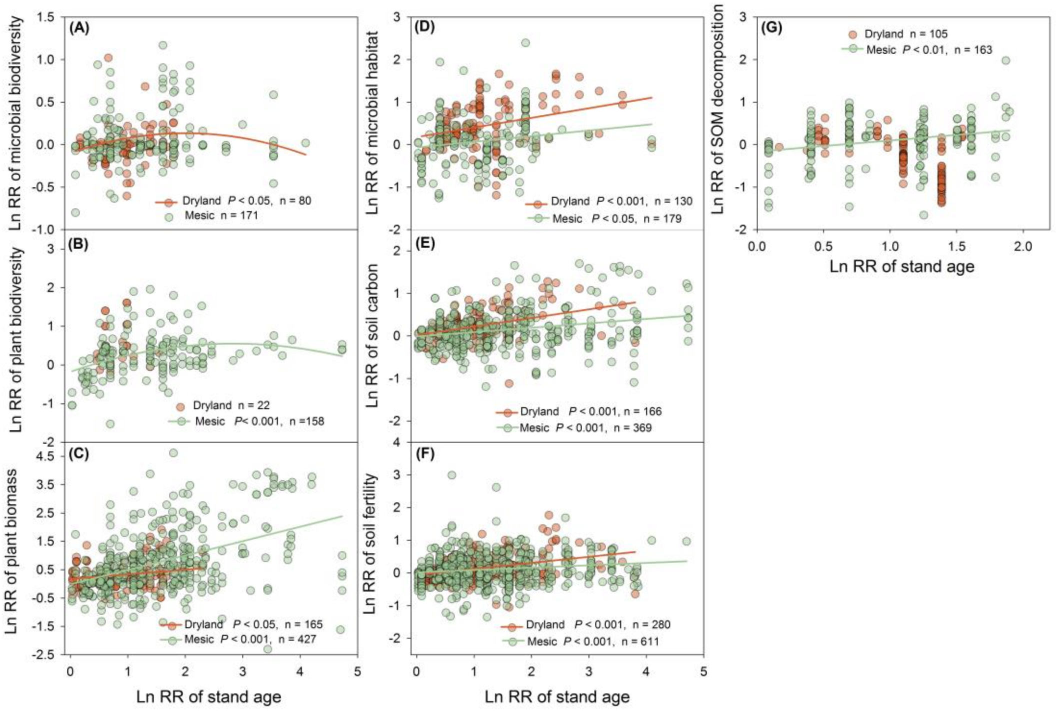

Figure S5 Relationships between stand age with the response ratios (LnRR) of plant biodiversity (A), microbial biodiversity (B), plant biomass (C), microbial habitat (D), soil carbon (E), soil fertility (F) and SOM decomposition (G) for dryland and mesic forests.

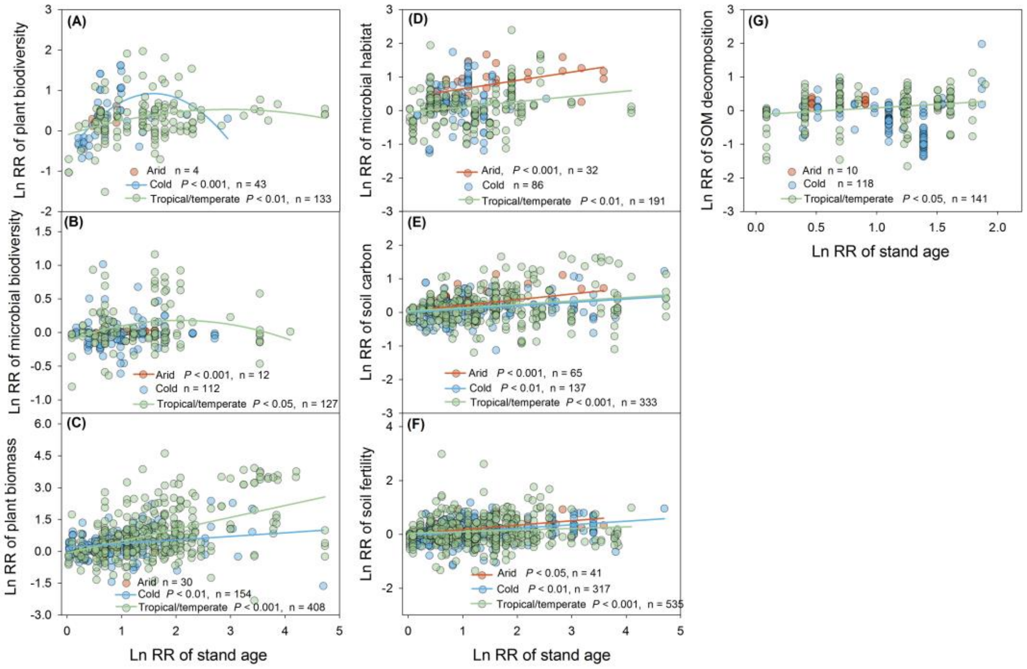

Figure S6 Relationships between stand age with the response ratios (LnRR) of plant biodiversity (A), microbial biodiversity (B), plant biomass (C), microbial habitat (D), soil carbon (E), soil fertility (F) and SOM decomposition (G) for arid, cold, and tropical/temperate forests.

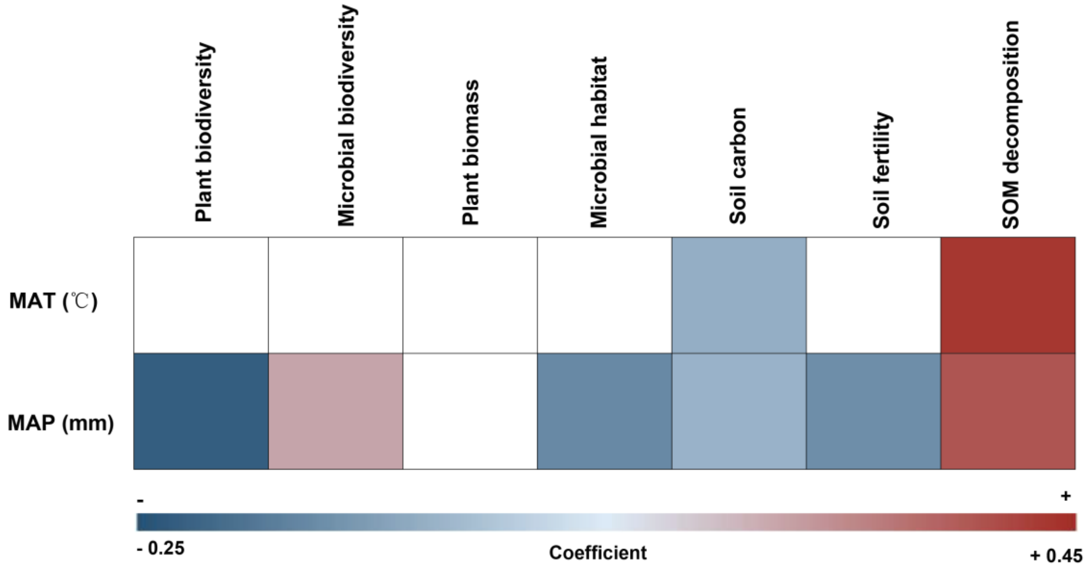

Figure S7 Partial correlation analyses reveals that climate is significantly correlated with the restoration of multiple ecosystem attributes (lnRR) even when controlling for changes across different age ranges (lnRR stand age).

Text S1 The reference lists of selected literature.

Zethof, J.H.T., Cammeraat, E.L.H., Nadal-Romero, M.E. The enhancing effect of afforestation over secondary succession on soil quality under semiarid climate conditions. *Science of the Total Environment*, **652**, 1090-1101(2018).

Jones, I. L., DeWalt, S. J., Lopez, O. R., Bunnefeld, L., Pattison, Z., Dent, D. H. Above- and belowground carbon stocks are decoupled in secondary tropical forests and are positively related to forest age and soil nutrients respectively. *Science of the Total Environment*, **697**, 133987 (2019).

Wang, X., Guo, Z., Guo, X., Wang, X. The Relative Importance of Succession, Stand Age and Stand Factors on Carbon Allocation of Korean Pine Forests in the Northern Mt. Xiaoxing'anling, China. *Forests*, **11**, 512 (2020).

Winbourne, J. B., Feng, A., Reynolds, L., Piotto, D., Hastings, M. G., Porder, S. Nitrogen cycling during secondary succession in Atlantic Forest of Bahia, Brazil. *Scientific Reports*, **8**, 1377 (2018).

Zhang-Turpeinen, H., Kivimäenpää, M., Aaltonen, H., Berninger, F., Köster, E., Köster, K. et al.Wildfire effects on BVOC emissions from boreal forest floor on permafrost soil in Siberia. *Science of the Total Environment*, **711**, 134851 (2020).

Lin, K. Accumulation of carbon, nitrogen, phosphorus, and fractionation of soil phosphorus in the subtropical human-assisted natural regeneration secondary *Castanopsis carlesii* forests. Ph.d dissertation for Fujian Normal University (2015).

Liu, J., Huang, B., Xu, Y., Lu, P., Lin, Y. Soil microbial functional diversity of typical Forest in Jigoshan Mountain Nature Reserve of Henan province. *Forest Resources Management*, **1**, 76-85 (2013).

Wu, R., Kang, F., Han, H., Cheng, X., Zhou, W., Wang, L. et al. Soil microbial properties in *Larix principis-rupprechtii* plantations of different ages in Mt. Taiyue, Shanxi,China. *Chinese Journal of Ecology*, **35**, 3183-3190 (2016).

Duo, W. The research on soil microbial biomass and diversity of 3 forests in subtropics.Master thesis for Central South University of Forestry and Technology (2012).

Xiong, X., Zhou, G., Zhang, D. Soil organic carbon accumulation modes between pioneer and old-growth forest ecosystems. *Journal of Applied Ecology*, **57**, 2419-2428 (2020).

Liu, C., Zuo, W., Zhao, Z., Qiu, L. Bacterial diversity of different successional stage forest soils in Dinghushan. *Acta Microbiologica Sinica*, **52**, 1489-1496 (2012).

Hume, A., Chen, H. Y. H., Taylor, A. R., Kayahara, G. J., Man, R. Soil C:N:P dynamics during secondary succession following fire in the boreal forest of central Canada. *Forest Ecology and Management*, **369**, 1-9 (2016).

Lu, H. Change characteristics of soil microbial diversity and its ecological function in *Pinus tabulaeformis* and *Larix kaempferi* Plantation in Weat Qinlung Mountains. Master thesis for Lanzhou University (2010).

Ren, C. Effects of plant-soil collaborative recovery and microbial responses in the loess plateau. Ph.d dissertation for Northwest A&F University (2018).

Chen, G., Yang, Z., Gao, R., Xie, J., Guo, J., Huang, Z., Yang, Y. Carbon storage in a chronosequence of Chinese fir plantations in southern China. *Forest Ecology and Management*, **300**, 68-76 (2013).

Tongkoom, K., Marohn, C., Piepho, H. P., Cadisch, G. Ecosystem recovery indicators as decision criteria on potential reduction of fallow periods in swidden systems of Northern Thailand. *Ecological Indicators*, **95**, 554-567 (2018).

Yuan, Z., Ali, A., Wang, S., Wang, X., Lin, F., Wang, Y. et al. Temporal stability of aboveground biomass is governed by species asynchrony in temperate forests. *Ecological Indicators,* **107**, 105661 (2019).

Wang, J., Cheng, Y., Zhang, J., Müller, C., Cai, Z. Soil gross nitrogen transformations along a secondary succession transect in the north subtropical forest ecosystem of southwest China. *Geoderma*, **280**, 88-95 (2016).

Zaitsev, A. S., Chauvat, M., Pflug, A., Wolters, V. Oribatid mite diversity and community dynamics in a spruce chronosequence. *Soil Biology and Biochemistry,* **34**, 1919-1927 (2002).

Yan, J., Zhang, D., Zhou, G., Liu, J. Soil respiration associated with forest succession in subtropical forests in Dinghushan Biosphere Reserve. *Soil Biology and Biochemistry*, **41**, 991-999 (2008).

Shao, S., Zhao, Y., Zhang, W., Hu, G., Xie, H., Yan, J. et al. Linkage of microbial residue dynamics with soil organic carbon accumulation during subtropical forest succession. *Soil Biology and Biochemistry,* **114**, 114-120 (2017).

Deng, L., Wang, K., Chen, M., Shangguan, Z., Sweeney, S. Soil organic carbon storage capacity positively related to forest succession on the Loess Plateau, China. *Catena*, **110**, 1-7 (2013).

Reyes, H.A., Ferreira, P.F.A., Silva, L.C. et al. Arbuscular mycorrhizal fungi along secondary forest succession at the eastern periphery of Amazonia: Seasonal variability and impacts of soil fertility. *Applied Soil Ecology*, **136**, 1-10 (2019).

Zhang, W., Qiao, W., Gao, D., Dai, Y., Deng, J., Yang, G. et al. Relationship between soil nutrient properties and biological activities along a restoration chronosequence of *Pinus tabulaeformis* plantation forests in the Ziwuling Mountains, China. *Catena*, **161**, 85-95 (2018).

Zhao, F. Z., Fan, X. D., Ren, C. J., Zhang, L., Han, X. H., Yang, G. H. et al. Changes of the organic carbon content and stability of soil aggregates affected by soil bacterial community after afforestation. *Catena*, **171**, 622-631 (2018).

Zhang, Y., Shangguan, Z. Interaction of soil water storage and stoichiometrical characteristics in the long-term natural vegetation restoration on the Loess Plateau. *Ecological Engineering*, **116**, 7-13 (2018).

Lu, M., Wang, S., Zhang, Z., Chen, M., Li, S., Cao, R., . . . Wang, P. Modifying effect of ant colonization on soil heterogeneity along a chronosequence of tropical forest restoration on slash-burn lands. *Soil & Tillage Research*, **194**, 104329 (2019).

Zhao, Y., Liu, X., Wang, Z., Zhao, S. Soil organic carbon fractions and sequestration across a 150-yr secondary forest chronosequence on the Loess Plateau, China. *Catena*, **133**, 303-308 (2015).

Mo, J., Brown, S., Peng, S., Kong, G. Nitrogen availability in disturbed, rehabilitated and mature forests of tropical China. *Forest Ecology and Management*, **175**, 573-583 (2003).

Dou, X., Deng, Q., Li, M., Wang, W., Zhang, Q., Cheng, X. Reforestation of *Pinus*

massoniana alters soil organic carbon and nitrogen dynamics in eroded soil in south China. *Ecological Engineering*, **52**, 154-160 (2013).

Powers, J.S., Becknell, J. M., Irving, J., Pèrez-Aviles, D. Diversity and structure of regenerating tropical dry forests in Costa Rica: Geographic patterns and environmental drivers. *Forest Ecology and Management*, **258**, 959-970 (2008).

Jia, G., Cao, J., Wang, C., Wang, G. Microbial biomass and nutrients in soil at the different stages of secondary forest succession in Ziwulin, northwest China. *Forest Ecology and Management*, **217**, 117-125 (2005).

Toniato, M.T.Z., Oliveira-Filho, A.Variations in tree community composition and structure in a fragment of tropical semideciduous forest in southeastern Brazil related to different human disturbance histories. *Forest Ecology and Management*, **198**, 319-339 (2004).

Liu, R. Effects of plant roots on soil carbon accumulation along subtropical evergreen forest successions. Ph.d dissertation for East China Normal University. (2019).

Shi, X. Soil microbial activity and community functional diversity in *Eucalyptus* platantion. Master thesis for Guangxi University. (2012).

Zhao, H., Zhou, Y., Ren Q. Evolution of soil microbial community structure and functional diversity in *Pinus Massoniana* plantations with age of stand. *Acta Pedologica Sinica*, **57**, 227-238 (2020).

Shi, L. Soil microbial diversity and comparative study of soil carbon and nitrogen pools of Chinese fir forest under different management. Master thesis for Fujian Agriculture and Forestry University (2017).

Cai, Z., Zhang, Y., Yang, C., Wang, S. Land-use type strongly shapes community composition, but not always diversity of soil microbes in tropical China. *Catena*, **165**, 369-380 (2018).

Guggenberger, G., Zech, W. Soil organic matter composition under primary forest, pasture, and secondary forest succession, Región Huetar Norte, Costa Rica. *Forest Ecology and Management*, **124**, 93-104 (1999).

Zhu, W., Cai, X., Liu, X., Wang, Ji., Cheng, S., Zhang, X., et al. Soil microbial population dynamics along a chronosequence of moist evergreen broad-leaved forest succession in southwestern China. *Journal of Mountain Science*, **7**, 327-338 (2010).

Liu, X., Lu, Y., Zhou, Y., Lei, X., Zhang, X., Meng, J. The influence of soil conditions on regeneration establishment for degraded secondary forest restoration, Southern China. *Forest Ecology and Management*, **261**, 1771-1780 (2011).

Amazonas, N.T., Martinelli, L.A., Piccolo, M., Rodrigues, R.R. Nitrogen dynamics during ecosystem development in tropical forest restoration. *Forest Ecology and Management*, **262**, 1551-1557 (2011).

Martelletti, S., Meloni, F., Freppaz, M., Viglietti, D., Lonati, M., Enri, S. R. et al. Effect of zeolitite addition on soil properties and plant establishment during forest restoration. *Ecological Engineering*, **132**, 13-22 (2019).

Wu, Y., Chen, W., Li, Q., Guo, Z., Li, Y., Zhao, Z. et al. Ecoenzymatic stoichiometry and nutrient limitation under a natural secondary succession of vegetation on the Loess Plateau, China. *Land Degradation & Development*, **32**, 1-11 (2020).

Wu, R., Meng, H., Wang, Y., Gu, J. Effects of reforestation on ammonia-oxidizing

microbial community composition and abundance in subtropical acidic forest soils. *Applied Microbiology and Biotechnology*, **102**, 5309-5322 (2018).

Jin, X., Liu, Y., Hu, W., Wang, G., Kong, Z., Wu, L., Ge, G. Soil bacterial and fungal communities and the associated nutrient cycling responses to forest conversion after selective logging in a subtropical forest of China. *Forest Ecology and Management*, **444**, 308-317 (2019).

Yan, E., Wang, X., Huang, J. Shifts in plant nutrient use strategies under secondary forest succession. *Plant and Soil*, **289**, 187-197 (2006).

Yan, E., Wang, X., Guo, M., Zhong, Q., Zhou, W., Li, Y. Temporal patterns of net soil N mineralization and nitrification through secondary succession in the subtropical forests of eastern China. *Plant and Soil*, **320**, 181-194 (2009).

Negrete-Yankelevich, S., Fragoso, C., Newton, A. C., Heal, O.W. Successional changes in soil, litter and macroinvertebrate parameters following selective logging in a Mexican Cloud Forest. *Applied Soil Ecology*, **35**, 340-355 (2007).

Yang, B., Qi, K., Bhusal, D. R., Huang, J., Chen, W., Wu, Q. et al. Soil microbial community and enzymatic activity in soil particle-size fractions of spruce plantation and secondary birch forest. *European Journal of Soil Biology*, **99**, 103196 (2020).

Zhang, H., Shi, L., Wen, D., Yu, K.Soil potential labile but not occluded phosphorus forms increase with forest succession. *Biology and Fertility of Soils*, **52**, 41-51 (2016).

Zeng, Z., Wang, S., Zhang, C., Tang, H., Li, X., Wu, Z., Luo, J. Soil microbial activity and nutrients of evergreen broad-leaf forests in mid-subtropical region of China. *Journal of Forestry Research*, **26**, 673-678 (2015).

Zeng, Z., Wang, S., Zhang, C., Chao, G., Hu, Q. Carbon storage in evergreen broad-leaf forests in mid-subtropical region of China at four succession stages. *Journal of Forestry Research*, **24**, 677-682 (2013).

Yao, X., Zeng, W., Zeng, H.,Wang, W. Soil microbial attributes along a chronosequence of Scots pine (*Pinus sylvestris* var. *mongolica*) plantations in northern China. *Pedosphere*, **30**, 433-442 (2020).

Saynes, V., Hidalgo, C., Etchevers, J. D., Campo, J. E. Soil C and N dynamics in primary and secondary seasonally dry tropical forests in Mexico. *Applied Soil Ecology*, **29**, 282-289 (2004).

He, F., Yang, B., Wang, H., Yan, Q., Cao, Y., He, X. Changes in composition and diversity of fungal communities along *Quercus mongolica* forests developments in Northeast China. *Applied Soil Ecology*, **100**, 162-171(2016).

Xu, X., Yang, B., Wang, H., Cao, Y., Li, K., Gao, S. Temperature sensitivity of soil heterotrophic respiration is altered by carbon substrate along the development of *Quercus Mongolica* forest in northeast China. *Applied Soil Ecology*, **133**, 52-61 (2018).

Chen, H., Gurmesa, G.A,, Liu, L., Zhang, T., Fu, S., Liu, Z. et al. Effects of litter manipulation on litter decomposition in a successional gradients of tropical forests in southern China. *PLoS One*, **9**, e99018 (2014).

Feudis, M.D., Falsone, G., Vianello, G., Antisari, L.V. Stable organic carbon pool rises in soil under chestnut (*Castanea sativa* Mill.) forest for timber production after 15 years since grafting onto satin-cut stumps. *International Journal of Environmental Quality*,

40, 1-10 (2020).

Wang, X., Lu, Y., Xing, H., Zeng, J., Xie, Y., Cai, D. et al. Effects of Close-to-Nature Conversion on *Pinus massoniana* Plantations at Different Stand Developmental Stages. *Tropical Conservation Science*, **11**, 1-16 (2018).

Huang, Y., Ai, X., Yao, L., Zang, R., Ding, Y., Huang, J. et al. Changes in the diversity of evergreen and deciduous species during natural recovery following clear-cutting in a subtropical evergreen-deciduous broadleaved mixed forest of central China. *Tropical Conservation Science*, **8**, 1033-1052 (2015).

Aravena, J.C., Carmona, M.R., Pérez, C. A., Armesto, J.J. Changes in tree species richness, stand structure and soil properties in a successional chronosequence in northern Chiloé Island, Chile. *Revista Chilena de Historia Natural*, **75**, 339-360 (2002).

Lucas-Borja, M. E., Delgado-Baquerizo, M. Plant diversity and soil stoichiometry regulates the changes in multifunctionality during pine temperate forest secondary succession. *Science of the Total Environment*, **697**, 134204 (2019).

Brearley, F. Q. Below-ground secondary succession in tropical forests of Borneo. *Journal of Tropical Ecology*, **27**, 413-420 (2011).

Zhang, K., Xu, X., Wang, Q., Liu, B. Biomass, and carbon and nitrogen pools in a subtropical evergreen broad-leaved forest in eastern China. *Journal of Forest Research*, **15**, 274-282 (2010).

Castellanos-Castro, C., Newton, A.C. Environmental heterogeneity influences successional trajectories in colombian seasonally dry tropical forests. *Biotropica*, **47**, 660-671 (2015).

Lu, X., Zang, R., Ding, Y., Huang, J. Changes in biotic and abiotic drivers of seedling species composition during forest recovery following shifting cultivation on Hainan Island, China. *Biotropica*, **48**, 758-769 (2016).

McClellan, M., Montgomery, R., Nelson, K., Becknell, J. Comparing forest structure and biodiversity on private and public land: secondary tropical dry forests in Costa Rica. *Biotropica*, **50**, 510-519 (2018).

Cao, J., Pan, H., Chen, Z., Shang, H. Bacterial, fungal, and archaeal community assembly patterns and their determining factors across three subalpine stands at different stages of natural restoration after clear-cutting. *Journal of Soils and Sediments*, **20**, 2794-2803 (2020).

Alberti, G., Peressotti, A., Piussi, P., Zerbi, G. Forest ecosystem carbon accumulation during a secondary succession in the Eastern Prealps of Italy. *Forestry*, **81**, 622736 (2008).

Lu, X., Zang, R., Ding, Y., Huang, J. Partitioning the functional variation of tree seedlings during secondary succession in a tropical lowland rainforest. *Ecosphere*, **9**, e02305 (2018).

Emilio, B., Giovanna, B., Luciano, G., Agata, N., Juliane, R., Giovanna, S. et al. Carbon stock increases up to old growth forest along a secondary succession in Mediterranean island ecosystems. *PLoS One*, **14**, e0220194 (2019).

Emmer, I. M., Fanta, J., Kobus, A. T., Kooijman, A., Sevink, J. Reversing borealization as a means to restore biodiversity in Central-European mountainforests – an example from the Krkonoše Mountains, Czech Republic. *Biodiversity and Conservation*, **7**, 229-247 (1998).

Bai, Z., Wu, X., Lin, J.J., Xie, H.T., Yuan, H.S., Liang, C. Litter-, soil- and C:N-stoichiometry-associated shifts in fungal communities along a subtropical forest succession. *Catena*, **178**, 350-358 (2019).

Fang, H., Yu, G., Cheng, S., Zhu, T., Zheng, J., Mo, J. et al. Nitrogen-15 signals of leaf-litter-soil continuum as a possible indicator of ecosystem nitrogen saturation by forest succession and N loads. *Biogeochemistry*, **102**, 251-263 (2011).

Kaneda, S., Angst, Š., Frouz, J. Development of nutrient uptake by understory plant *Arrhenatherum elatius* and microbial biomass during primary succession of forest soils in post-mining land. *Forests*, **11**, 247 (2020).

Smith, A. P., Marín-Spiotta, E., Balser, T. Successional and seasonal variations in soil and litter microbial community structure and function during tropical postagricultural forest regeneration: a multiyear study. *Global Change Biology*, **21**, 3532-3547 (2015).

Han, T., Ren, H., Wang, J., Lu, H., Song, G., Chazdon, R. L. Variations of leaf eco-physiological traits in relation to environmental factors during forest succession. *Ecological Indicators*, **117**, 106511(2020).

Li, H., Wang, X., Liang, C., Hao, Z., Zhou, L., Ma, S. et al. Aboveground-belowground biodiversity linkages differ in early and late successional temperate forests. *Scientific Reports*, **5**, 12234 (2015).

Han, T., Huang, W., Liu, J., Zhou, G., Xiao, Y. Different soil respiration responses to litter manipulation in three subtropical successional forests. *Scientific Reports*, **5**, 18166 (2015).

Bush, J. K. Soil nitrogen and carbon after twenty years of riparian forest development. *Soil Science Society of America Journal*, **72**, 815-822 (2008).

Tang, J., Cao, M., Zhang, J., Li, M. Litterfall production, decomposition and nutrient use efficiency varies with tropical forest types in Xishuangbanna, SW China: a 10-year study. *Plant and Soil*, **335**, 271-288(2010).

Tang, C. Q., Li, Y.-H., Zhang, Z.-Y., Hou, X., Hara, K., Tomita, M. et al. Effects of management on vegetation dynamics and associated nutrient cycling in a karst area, Yunnan, SW China. *Landscape and Ecological Engineering*, **11**, 177-188 (2015).

Valdespino, P., Romualdo, R., Cadenazzi, L., Campo, J. Phosphorus cycling in primary and secondary seasonally dry tropical forests in Mexico. *Annals of Forest Science*, **66**, 107(2009).

Hasselquist, N. J., Allen, M. F., Santiago, L.S. Water relations of evergreen and drought-deciduous trees along a seasonally dry tropical forest chronosequence. *Oecologia*, 164, 881-890 (2010).

Huang, W., Liu, J., Wang, Y. P., Zhou, G., Han, T., Li, Y. Increasing phosphorus limitation along three successional forests in southern China. *Plant and Soil*, **364**, 181-191(2013).

Yuan, Z.Y., Chen, H.Y.H. Fine root dynamics with stand development in the boreal forest. *Functional Ecology*, **26**, 991-998 (2012).

Aidar, M. P. M., Schmidt, S., Moss, G., Stewart, G. R., Joly, C.A. Nitrogen use strategies of neotropical rainforest trees in threatened Atlantic Forest. *Plant, Cell & Environment*, **26**, 389-399 (2003).

Liu, S. L., Fu, B. J., Lü, Y. H., Chen, L.D. Effects of reforestation and deforestation on soil

properties in humid mountainous areas: a case study in Wolong Nature Reserve, Sichuan province, China. *Soil Use and Management*, **18**, 376-380 (2002).

Li, Y., Yang, F., Ou, Y., Zhang, D., Liu, J., Chu, G. et al. Changes in forest soil properties in different successional stages in lower tropical China. *PLoS One*, **8**, e81359 (2013).

Dang, P., Yu, X., Le, H., Liu, J., Shen, Z., Zhao, Z. Effects of stand age and soil properties on soil bacterial and fungal community composition in Chinese pine plantations on the Loess Plateau. *PLoS One*, **12**, e0186501(2017).

Chen, X., Li, Y., Mo, J., Otieno, D., Tenhunen, J., Yan, J. et al. Effects of nitrogen deposition on soil organic carbon fractions in the subtropical forest ecosystems of S China. *Journal of Plant Nutrition And Soil Science*, **175**, 947-953 (2012).

Woodbury, D. J., Yassir, I., Arbainsyah, Doroski, D. A., Queenborough, S. A., Ashton, M. S. Filling a void: Analysis of early tropical soil and vegetative recovery under leguminous, post-coal mine reforestation plantations in East Kalimantan, Indonesia. *Land Degradation & Development*, **31**, 473-487 (2020).

Liu, J., Jia, X., Yan, W., Zhong, Y., Shangguan, Z. Changes in soil microbial community structure during long-term secondary succession. *Land Degradation & Development*, **31**, 1151-1166 (2020).

Liu, B., Biswas, S. R., Yang, J., Liu, Z., He, H. S., Liang, Y. et al. Strong influences of stand age and topography on post-fire understory recovery in a Chinese boreal forest. *Forest Ecology and Management*, **473**, 118307 (2020).

Liu, G., Chen, L., Shi, X., Yuan, Z., Yuan, L.Y., Lock, T.R., Kallenbach, R.L. Changes in rhizosphere bacterial and fungal community composition with vegetation restoration in planted forests. *Land Degradation & Development*, **30**, 1147-1157(2019).

Liu, Y., Zhu, G., Hai, X., Li, J., Shangguan, Z., Peng, C., Deng, L. Long-term forest succession improves plant diversity and soil quality but not significantly increase soil microbial diversity: Evidence from the Loess Plateau. *Ecological Engineering*, **142**, 105631 (2020).

Luan, J., Liu, S., Zhu, X., Wang, J. Soil carbon stocks and fluxes in a warm-temperate oak chronosequence in China. *Plant and Soil*, **347**, 243-253 (2011).

Bauters, M., Vercleyen, O., Vanlauwe, B., Six, J., Bonyoma, B., Badjoko, H. et al. Long-term recovery of the functional community assembly and carbon pools in an African tropical forest succession. *Biotropica*, **51**, 319-329 (2019).

Ostertag, R., Marín-Spiotta, E., Silver, W. L., Schulten, J. Litterfall and decomposition in relation to soil carbon pools along a secondary forest chronosequence in Puerto Rico. *Ecosystems*, **11**, 701-714(2008).

Negrete-Yankelevich, S. et al. Decomposition and macroinvertebrates in experimental litter along a secondary chronosequence of tropical montane forest. *Biology and Fertility of Soils*, **44**, 853-861(2008).

Pereira, N. A., Prima, S. D., Bovi, R. C., Silva, L. F. S. d., Godoy, G. d., Naves, R. P., Cooper, M. Does the Process of Passive Forest Restoration Affect the Hydrophysical Attributes of the Soil Superficial Horizon? *Water*, **12**, 1689 (2020).

Pérez, C.A., Carmona, M.R., Aravena, J.C., Armesto, J.J. Successional changes in soil nitrogen availability, non-symbiotic nitrogen fixation and carbon/nitrogen ratios in southern Chilean forest ecosystems. *Oecologia*, **140**, 617-625(2004).

Perakis, S.S., Tepley, A.J., Compton, J.E. Disturbance and topography shape nitrogen availability and $\delta^{15}N$ over long-term forest succession. *Ecosystems*,**18**, 573-588(2015).

Ma, R., Hu, F., Liu, J., Wang, C., Wang, Z., Liu, G., Zhao, S. Shifts in soil nutrient concentrations and C:N:P stoichiometry during long-term natural vegetation restoration. *Peer J*, **8**, e8382(2020).

Shao, P., Liang, C., Rubert-Nason, K., Li, X., Xie, H., Bao, X. Secondary successional forests undergo tightly-coupled changes in soil microbial community structure and soil organic matter. *Soil Biology and Biochemistry*, **128**, 56-65 (2019).

Schwendenmann, L. Soil properties of boreal riparian plant communities in relation to natural succession and clear-cutting, peace river lowlands, Wood Buffalo National Park, Canada. *Water, Air, and Soil Pollution*, **122**, 449-467(2000).

Feng, Y., Han, S., Chen, W., Gu, Y., Stewart, C. E., Zhang, J. et al. Variation in soil lignin protection mechanisms in five successional gradients of mixed broadleaf–pine forests. *Soil Science Society of America Journal*, **84**, 232-250 (2020).

Yassir, I., Peter, B. Soil organic matter dynamics upon secondary succession in Imperata Grassland, East Kalimantan, Indonesia. *Indonesian Journal of Forestry Research*, **2**, 43-53(2015).

Song, P., Ren, H., Jia Q., Guo, J., Zhang, N., Ma, K. Effects of historical logging on soil microbial communities in a subtropical forest in southern China. *Plant and Soil*, **397**, 115-126(2015).

Huang, Y., Zhang, X., Zang, R., Fu, S., Ai, X., Yao, L.et al. Functional recovery of a subtropical evergreen-deciduous broadleaved mixed forest following clear cutting in central China. *Scientific Reports*, **8**, 16458(2018).

Winbourne, J. B., Feng, A., Reynolds, L., Piotto, D., Hastings, M. G., Porder, S. Nitrogen cycling during secondary succession in Atlantic Forest of Bahia, Brazil. *Scientific Reports*, **8**, 1377(2018).

Sokołowska, J., Józefowska, A., Woźnica, K., Zaleski, T. Succession from meadow to mature forest: Impacts on soil biological, chemical and physical properties—Evidence from the Pieniny Mountains, Poland. *Catena*, **189**, 104503(2020).

Mylliemngap, W., Nath, D., Barik, S.K. Changes in vegetation and nitrogen mineralization during recovery of a montane subtropical broadleaved forest in North-eastern India following anthropogenic disturbance. *Ecological Research*, **31**, 21-38 (2016).

Tang, C.Q., Zhao, M. H., Li, X.S., Ohsawa, M., Ou, X.K. Secondary succession of plant communities in a subtropical mountainous region of SW China. *Ecological Research*, **25**, 149-161(2010).

Wang, C., Ma, Y., Trogisch, S., Huang, Y., Geng, Y., Scherer-Lorenzen, M., He, J. Soil respiration is driven by fine root biomass along a forest chronosequence in subtropical China. *Journal of Plant Ecology*, **10**, 36-46 (2017).

Mo, J., Brown, S., Xue, J., Fang, Y., Li, Z. Response of litter decomposition to simulated N deposition in disturbed, rehabilitated and mature forests in subtropical China. *Plant and Soil*, **282**, 135-151(2006).

Yan, B., Sun, L., Li, J., Liang, C., Wei, F., Xue, S., Wang, G. Change in composition and potential functional genes of soil bacterial and fungal communities with secondary succession in *Quercus liaotungensis* forests of the Loess Plateau, western China.

Geoderma, **364**, 114199 (2020).
Wang, J. Y., Ren, C. J., Feng, X. X., Zhang, L., Doughty, R., Zhao, F. Z.Temperature sensitivity of soil carbon decomposition due to shifts in soil extracellular enzymes after afforestation. *Geoderma*, **374**, 114426 (2020).
Wang, H., Bu, L., Song, F., Tian, J.,Wei, G. Soil available nitrogen and phosphorus affected by functional bacterial community composition and diversity as ecological restoration progressed. *Land Degradation & Development*, **32**, 1-16 (2020).
Yan, W., Zhong, Y., Zhu, G., Liu, W., Shangguan, Z. Nutrient limitation of litter decomposition with long-term secondary succession: evidence from controlled laboratory experiments. *Journal of Soils and Sediments*, **20**, 1858-1868 (2020).
